# Nucleic DHX9 cooperates with STAT1 to transcribe interferon-stimulated genes

**DOI:** 10.1126/sciadv.add5005

**Published:** 2023-02-03

**Authors:** Xingxing Ren, Decai Wang, Guorong Zhang, Tingyue Zhou, Zheng Wei, Yi Yang, Yunjiang Zheng, Xuqiu Lei, Wanyin Tao, Anmin Wang, Mingsong Li, Richard A. Flavell, Shu Zhu

**Affiliations:** ^1^ Department of Digestive Disease, Division of Life Sciences and Medicine, The First Affiliated Hospital of University of Science and Technology of China, University of Science and Technology of China, 230001 Hefei, China.; ^2^ Institute of Immunology and the CAS Key Laboratory of Innate Immunity and Chronic Disease, Division of Life Sciences and Medicine, University of Science and Technology of China, Hefei 230027, China.; ^3^ Department of Gastroenterology, Third Affiliated Hospital of Guangzhou Medical University, 510145 Guangzhou, China.; ^4^ Department of Immunobiology, Yale University School of Medicine, 300 Cedar Street, New Haven, CT 06510, USA.; ^5^ Howard Hughes Medical Institute, Yale University School of Medicine, 300 Cedar Street, New Haven, CT 06510, USA.; ^6^ School of Data Science, University of Science and Technology of China, Hefei 230026, China.; ^7^ Institute of Health and Medicine, Hefei Comprehensive National Science Center, Hefei, China.

## Abstract

RNA helicase DHX9 has been extensively characterized as a transcriptional regulator, which is consistent with its mostly nucleic localization. It is also involved in recognizing RNA viruses in the cytoplasm. However, there is no in vivo data to support the antiviral role of DHX9; meanwhile, as a nuclear protein, if and how nucleic DHX9 promotes antiviral immunity remains largely unknown. Here, we generated myeloid-specific and hepatocyte-specific DHX9 knockout mice and confirmed that DHX9 is crucial for host resistance to RNA virus infections in vivo. By additional knockout MAVS or STAT1 in DHX9-deficient mice, we demonstrated that nucleic DHX9 plays a positive role in regulating interferon-stimulated gene (ISG) expression downstream of type I interferon. Mechanistically, upon interferon stimulation, DHX9 is directly bound to STAT1 and recruits Pol II to the ISG promoter region to participate in STAT1-mediated transcription of ISGs. Collectively, these findings uncover an important role for nucleic DHX9 in antiviral immunity.

## Teaser

Nucleic DHX9 Promotes the Antiviral Immune Response via Facilitating STAT1-Mediated Transcription of Interferon-Stimulated Genes.

## INTRODUCTION

Innate immune response is the first line of host defense against infection and attacks from invading pathogens. Upon recognizing pathogen-associated molecular patterns, host-encoded pattern recognition receptors (PRRs) induce the transcription of genes encoding inflammatory cytokines and interferons (IFNs) to eliminate microbes (*1*). During infection with RNA viruses, Toll-like receptors and RNA helicase retinoic acid–inducible gene I (RIG-I)–like receptors sense viral nucleic acids and activate IFN-regulatory factors (IRFs) and nuclear factor κB (NF-κB) to trigger the production of type I IFNs and proinflammatory cytokines (*2*, *3*). Type I IFN is critical for the innate immune response against viral infections, which induces many multifunctional IFN-stimulated genes (ISGs) through the Janus kinase–signal transducer and activator of transcription (JAK-STAT) signaling pathway (*4*). However, the mechanisms underlying the regulation of type I IFN–triggered STAT signaling and ISG expression have not been fully elucidated (*5*).

DExD/H-box (DDX/DHX) RNA helicases play important roles in antiviral signaling. RIG-I (also named DDX58), melanoma differentiation-associated protein 5 (MDA5) (also named Helicard), andRIG-I-like receptor LGP2 (also named DHX58)are part of the DExD/H RNA helicase family and are one of the most important groups of antiviral PRRs (*6*–*8*). DDX3 binds to the kinases TBK1 and inhibitor of NF-κB kinase ɛ (IKKɛ) to enhance IFN-β production (*9*). DHX9 (*10*), DHX15 (*11*, *12*), DHX29 (*13*), and DHX33 (*14*) directly recognize double-stranded RNA (dsRNA) and activate antiviral signaling. These findings indicate that DExD/H-box RNA helicases regulate antiviral immune responses in multiple layers. However, most of the DExD/H-box family members are located in the nucleus and control nearly every aspect of RNA metabolism (*15*); therefore, it is reasonable to speculate that RNA helicases play a nucleic role in regulating antiviral innate immune responses.

DHX9 or RNA helicase A widely participates in RNA processing (*16*), transcription (*17*, *18*), and translation (*19*). Because of its pleiotropic functions, DHX9 has also been identified as a sensor for dsRNA or C-phosphate-G (CpG) DNA in myeloid cells linked to innate immunity (*10*, *20*). Our previous studies showed that DHX9 pairs with Nlrp9b to sense short dsRNA and forms inflammasome complexes with the adaptor proteins Asc and caspase-1 to promote the maturation of interleukin-18 and Gsdmd-induced pyroptosis and resist rotavirus infection in intestinal epithelial cells (IECs) (*21*). In addition, studies have shown that DHX9 can bind to the NF-κB P65 subunit in the nucleus to promote transcription of downstream proinflammatory cytokines that can resist DNA virus infections (*22*). Many studies have demonstrated that a broad range of viruses hijacks DHX9 to evade the innate immune system and promote self-replication, such as HIV-1 (*23*), hepatitis B virus (*24*), Myxoma virus (*25*), and Chikungunya virus (*26*). Therefore, the role of DHX9 in the interaction between the host’s innate immune system and viruses remains unclear, and in vivo data are necessary to explore the specific functions of DHX9.

DHX9 is a multifunctional nuclear protein, and it is unclear whether it plays a role in regulating innate immunity against RNA viruses in the nucleus. This study demonstrates that DHX9 is intimately involved in innate immune responses against RNA virus infection in vivo. In addition to sensing RNA in the cytoplasm, DHX9 interacts with STAT1 in the nucleus, which acts as a transcriptional regulator to facilitate the transcription of downstream ISGs. Hence, our study highlights the nucleic function of DHX9 in controlling the IFN-STAT1-ISG cascade.

## RESULTS

### DHX9 is essential for host resistance to RNA virus infection

**Fig. 1.**
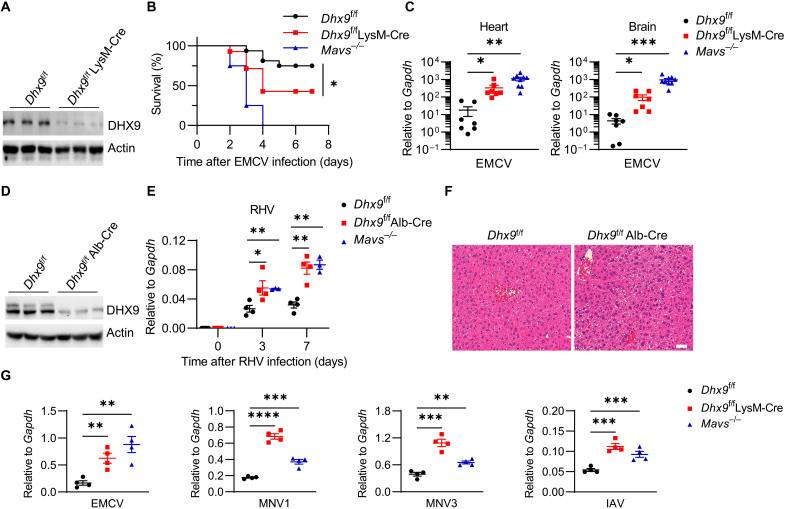
DHX9 is essential for host defense against RNA virus in vivo and in vitro. (**A**) DHX9 deficiency in BMDMs from *Dhx9*^f/f^ LysM-Cre mice was confirmed by Western blotting. Data are shown as representative of three independent experiments. (**B**) Survival rates of age- and sex-matched *Dhx9*^f/f^ mice (*n* = 16), *Dhx9*^f/f^ LysM-Cre mice (*n* = 14), and *Mavs*^−/−^ mice (*n* = 12) after intraperitoneal injection with 1000 plaque-forming units (PFU) of EMCV virus. (**C**) RT-qPCR of mRNA expression for EMCV replicase in the heart and brain tissues from *Dhx9*^f/f^, *Dhx9*^f/f^ LysM-Cre, and *Mavs*^−/−^ mice at 3 days after intraperitoneal injection with 1000 PFU EMCV (*n* ≥ 7). (**D**) DHX9 deficiency in parenchymal hepatic cells from *Dhx9*^f/f^ Alb-Cre mice was confirmed by Western blotting. Data are shown as representative of three independent experiments. (**E**) RHV mRNA transcripts were analyzed by RT-qPCR in the livers from *Dhx9*^f/f^ (*n* = 11), *Dhx9*^f/f^ LysM-Cre (*n* = 11), and *Mavs*^−/−^ mice (*n* = 9) with RHV infection by intravenous injection (2 × 10^7^ PFU/g body weight). (**F**) Hematoxylin and eosin staining of liver sections from mice described in (E). Scale bar, 100 μm. (**G**) BMDMs from *Dhx9*^f/f^, *Dhx9*^f/f^ LysM-Cre, and *Mavs*^−/−^ mice were infected with EMCV, MNV1, MNV3, or IAV; after 12 hours, the cells were collected for RT-qPCR of mRNA expression for viruses replicase. Data represent four independent experiments. Data are shown as means ± SEM and were analyzed by Kaplan-Meier analysis (B) and two-tailed unpaired Student’s *t* test (C, E, and G); *P < 0.05, **P < 0.01, ***P < 0.001, and ****P < 0.0001.

Previous studies have suggested that DHX9 is involved in resistance to RNA viral infections in plasmacytoid dendritic cells and IECs (*10*, *21*); however, in vivo data supporting the universal anti-RNA virus role of DHX9 are still lacking. To investigate the effects of DHX9 in innate antiviral immunity in vivo, we generated DHX9 conditional knockout mice that specifically targeted deletion in macrophages by crossing *Dhx9*^f/f^ mice with LysM-Cre (express Cre recombinase under the control of lysozyme 2 promoter) transgenic mice (fig. S1A) and confirmed the successful deficiency of DHX9 in *Dhx9*^f/f^ LysM-Cre mouse macrophages (Fig. 1A). We first evaluate the myeloid development in these mice. The proportion of myeloid cells in the spleen, bone marrow, and mesenteric lymph nodes was comparable between *Dhx9*^f/f^ littermates and *Dhx9*^f/f^ LysM-Cre mice (fig. S1, B and C). There was also no notable difference in the proportion of peritoneal macrophages between *Dhx9*^f/f^ LysM-Cre mice and *Dhx9*^f/f^ mice (fig. S2, A to C). Then, we checked whether the macrophages are still functional after DHX9 depletion. We measured the bacterial phagocytic capacity of bone marrow–derived macrophages (BMDMs) from *Dhx9*^f/f^ and *Dhx9*^f/f^ LysM-Cre mice by bacterial phagocytosis assay using enhanced green fluorescent protein (GFP)–*Escherichia coli*. The results showed that the cellular fluorescence levels in *Dhx9*^f/f^ and *Dhx9*^f/f^ LysM-Cre BMDMs were comparable (fig. S3, A to C). Thus, DHX9 deficiency in the myeloid lineage does not affect the development, maturation, and phagocytic function of macrophages. Next, we evaluated the antiviral role of DHX9 in vivo. We infected mice with encephalomyocarditis virus (EMCV) by intraperitoneal injection. Both *Dhx9*^f/f^ LysM-Cre mice and positive control *Mavs*^−/−^ mice showed higher sensitivity to EMCV virus infection in the overall survival assay than *Dhx9*^f/f^ mice (Fig. 1B). We then performed a virological analysis of heart and brain tissues. The viral load was significantly higher in *Mavs*^−/−^ and *Dhx9*^f/f^ LysM-Cre mice than in *Dhx9*^f/f^ mice (Fig. 1C). Notably, there was no difference in the proportion of peritoneal macrophages between *Dhx9*^f/f^ and *Dhx9*^f/f^ LysM-Cre mice 2 days after EMCV infection, indicating that the resistance of DHX9 to EMCV infection was not determined by affecting the number of macrophages (fig S2, A to C), further confirming the antiviral role of DHX9 in vivo without affecting myeloid development.

To confirm the susceptible phenotype of DHX9-deficient mice to RNA viral infections in another organ, we generated DHX9 conditional knockout mice with a specifically targeted deletion in the hepatocytes, by crossing *Dhx9*^f/f^ mice with Alb-Cre (express Cre recombinase under the control of the mouse albumin promoter) transgenic mice and confirmed the successful deficiency of DHX9 in hepatocytes from *Dhx9*^f/f^ Alb-Cre mice (Fig. 1D). We infected mice with rat hepatitis virus [RHV; a single-stranded RNA (ssRNA) virus] by intravenous injection. Mouse liver tissue samples were collected at 3 and 7 days after infection. The viral loads in the liver from *Dhx9*^f/f^ Alb-Cre mice and positive control *Mavs*^−/−^ mice were significantly higher than that from *Dhx9*^f/f^ mice (Fig. 1E). Consistent with the viral loads, *Dhx9*^f/f^ Alb-Cre mice showed much stronger inflammatory cell infiltration in the hematoxylin and eosin staining of the liver in comparison to that of the littermate control mice (Fig. 1F). Therefore, DHX9 is also required for hepatic resistance to RHV infection in vivo.

To further test whether DHX9 is widely involved in the immune response against different kinds of RNA viral infections in vitro, we prepared the BMDMs from *Dhx9*^f/f^ LysM-Cre mice, littermate control *Dhx9*^f/f^ mice, and positive control *Mavs*^−/−^ mice. We then infected these BMDMs with dsRNA virus EMCV, two strains of murine norovirus (MNV) (positive-sense ssRNA virus), which cause acute infection (MNV1) or chronic infection (MNV3) (*27*), or a negative-sense ssRNA virus influenza A virus (IAV) in serum-free media for 12 hours. Consistent with the in vivo results, DHX9 or mitochondrial antiviral-signaling protein (MAVS) deficiency showed increased viral loads for different RNA viruses in BMDMs (Fig. 1G). Together, these data suggest that DHX9 protects against multiple RNA virus infections in different cells and organs.

### DHX9 deficiency dampens ISG induction

**Fig. 2.**
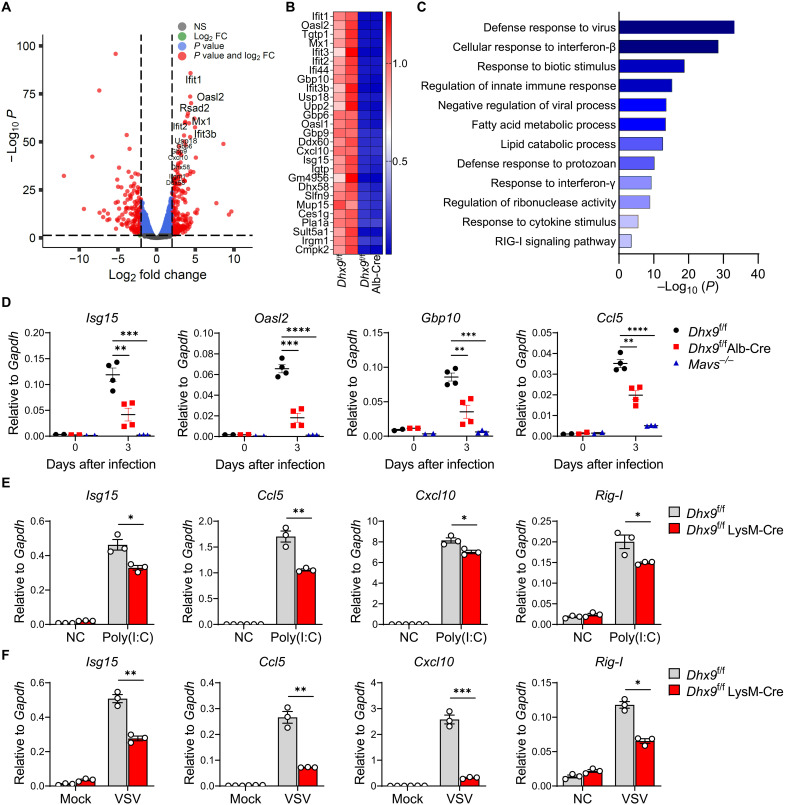
DHX9 deficiency dampens the ISG induction. (**A**) Volcano plot of genes differentially expressed (false discovery rate < 0.05) in the livers according to RNA-seq data of *Dhx9*^f/f^ mice compared to *Dhx9*^f/f^ Alb-Cre mice, which are infected with RHV at 3 days after infection (The gray dots represent genes with no statistically significant differences (NS); the red dots and the blue dots, *P* < 0.05; right-tailed Fischer’s exact *t* test). (**B**) Heatmap showing the relative expression value (*Z* score) of top genes according to RNA-seq data ranked by fold change (FC). (**C**) Gene ontology analysis of differentially expressed genes in *Dhx9*^f/f^ mouse liver compared to *Dhx9*^f/f^ Alb-Cre mice according to RNA-seq data (*P* < 0.05; right-tailed Fischer’s exact *t* test). (**D**) RT-qPCR of mRNA expression of *Isg15*, *Oasl2*, *Gbp10*, and *Ccl5* in the livers from *Dhx9*^f/f^ (*n* = 6), *Dhx9*^f/f^ LysM-Cre (*n* = 6), and *Mavs*^−/−^ mice (*n* = 5) with RHV infection, shown as means ± SEM. (**E**) BMDMs from *Dhx9*^f/f^ and *Dhx9*^f/f^ LysM-Cre mice were stimulated with poly(I:C) (5 μg/ml) for 6 hours, and the cells were collected for RT-qPCR of mRNA expression of *Isg15*, *Ccl5*, *Cxcl10*, and *Rig-I*. Data represent means ± SEM of three independent experiments. (**F**) BMDMs from *Dhx9*^f/f^ and *Dhx9*^f/f^ LysM-Cre mice were stimulated with VSV-GFP [multiplicity of infection (MOI) = 10] for 6 hours, and the cells were collected for RT-qPCR of mRNA expression of *Isg15*, *Ccl5*, *Cxcl10*, and *Rig-I*. Data represent means ± SEM of three independent experiments. Statistical analysis in (D), (E), and (F) was performed by two-tailed unpaired Student’s *t* test; *P < 0.05, **P < 0.01, ***P < 0.001, and ****P < 0.0001.

To get the mechanistic insight into the role of DHX9 in the host antiviral response, we performed RNA sequencing (RNA-seq) to examine transcriptomes of RHV-infected livers from *Dhx9*^f/f^ Alb-Cre and EMCV-infected peritoneal macrophages from *Dhx9*^f/f^ LysM-Cre mice together with their controls. In RHV-infected livers, a total of 1485 differentially expressed genes were enriched, and genes with the most significantly decreased expression in *Dhx9*^f/f^ Alb-Cre mice were a subset of ISGs, such as *Ifit1*, *Oasl2*, *Rsad2*, and *Mx1* (Fig. 2, A and B). Gene ontology analysis also revealed that the most highly enriched down-regulated pathways in the liver from *Dhx9*^f/f^ Alb-Cre mice compared to that from littermate control mice are associated with innate antiviral responses to viral infections, such as “defense response to virus,” “cellular response to IFN-β,” and “regulation of innate immune response” (Fig. 2C). The expression of certain altered ISGs, including *Isg15*, *Oasl2*, *Gbp10*, and *Ccl5*, was confirmed by reverse transcription quantitative polymerase chain reaction (RT-qPCR) analysis. Consistent with the results of RNA-seq, ISGs showed significantly decreased expression in the liver of *Dhx9*^f/f^ Alb-Cre mice or positive control *Mavs*^−/−^ mice compared to *Dhx9*^f/f^ mice (Fig. 2D). Similarly, we enriched peritoneal macrophages from both *Dhx9*^f/f^ LysM-Cre mice and *Dhx9*^f/f^ littermate control mice at 16 hours after EMCV infection followed by RNA-seq analysis. Gene ontology analysis revealed that the most highly enriched down-regulated pathways in peritoneal macrophages from *Dhx9*^f/f^ LysM-Cre were associated with defense response to viral infections, in particular, the IFN-ISG pathways, such as “defense response to virus,” “response to IFN-β,” and “positive regulation of response to cytokine stimulus” (fig. S4A). These transcriptome data indicated that depletion of DHX9 in macrophages results in a defect in innate immune responses to viruses. We also measured the expression levels of ISGs in the heart and brain tissues during the early stage of EMCV infection. ISGs were also significantly reduced in these tissues from *Dhx9*^f/f^ LysM-Cre mice (fig. S4B). Together, these data suggest that DHX9 plays an important role in regulating ISG expression in RNA virus infections in vivo.

We then explored the effect of DHX9 depletion on the expression of host antiviral genes in BMDMs. *Dhx9*^f/f^ and *Dhx9*^f/f^ LysM-Cre BMDMs were stimulated with ssRNA virus, vesicular stomatitis virus (VSV), or polyinosine-polycytidylic acid [poly(I:C)], which mimic dsRNA from the virus. BMDMs were collected at 6 hours after infection or stimulation to detect the expression of antiviral effector genes. The induction of ISGs, including *Isg15*, *Ccl5*, *Cxcl10*, and *Rig-1*, significantly decreased in BMDMs from *Dhx9*^f/f^ LysM-Cre mice compared to that in *Dhx9*^f/f^ mice (Fig. 2, E and F). Collectively, these data demonstrate that DHX9 is a positive regulator of ISG expression in response to RNA virus infection in cells and mice.

### The resistance of DHX9 to RNA virus infection is partially independent of its RNA sensing function

**Fig. 3.**
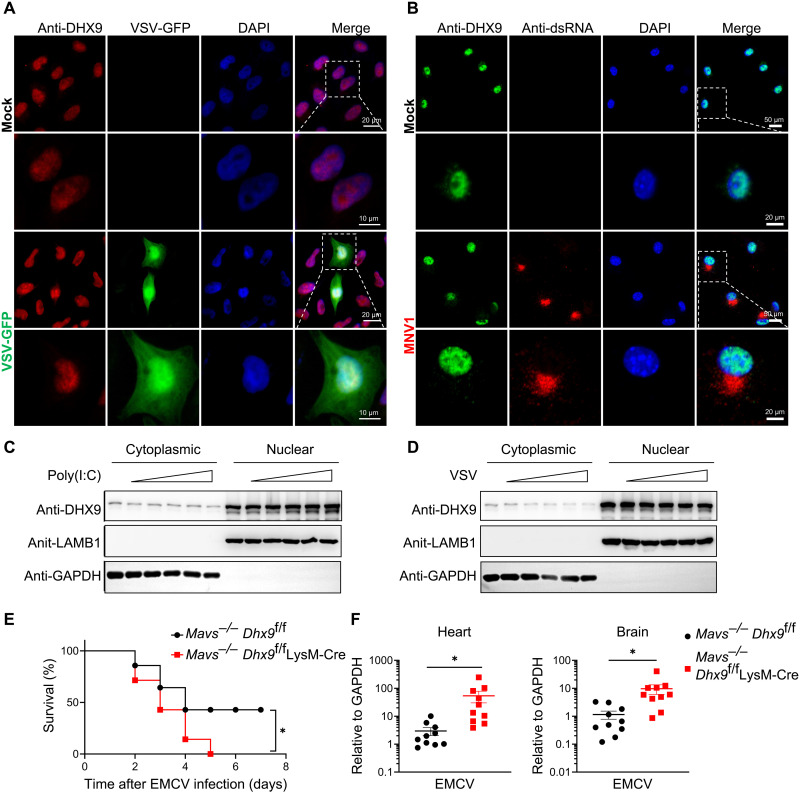
The resistance of DHX9 to RNA virus infection is partially independent of its RNA sensing function. (**A**) Representative confocal microscopy images of Hela cells that were either VSV-GFP–infected (bottom) or mock-infected (top) at MOI 5 for 12 hours and labeled with antibodies to the appropriate protein. Cells nuclei were visualized with 4,6-diamidino-2-phenylindole (DAPI). Scale bars, 10 and 20 μm. (**B**) Representative confocal microscopy images of BMDMs that were either MNV1-infected (bottom) or mock-infected (top) at MOI 1 for 12 hours. dsRNA was labeled by immunostaining with a mouse monoclonal antibody J2, and cell nuclei were visualized with DAPI. Scale bars, 20 and 50 μm. (**C**) Hela cells were stimulated with poly(I:C) (from 5 to 50 μg/ml) for 8 hours, cells were harvested and subjected to nuclear and cytoplasmic protein extraction, and Western blotting was used to detect the protein distribution of DHX9. LAMB1 and GAPDH were used as internal references. Data are shown as representative of three independent experiments. (**D**) Hela cells were infected with VSV (from 1 MOI to 10 MOI) for 8 hours, cells were harvested and subjected to nuclear and cytoplasmic protein extraction, and Western blotting was used to detect the protein distribution of DHX9. LAMB1 and GAPDH were used as internal references. Data are shown as representative of three independent experiments. (**E**) Survival rates of age- and sex-matched *Mavs*^−/−^ *Dhx9*^f/f^ and *Mavs*^−/−^ *Dhx9*^f/f^ LysM-Cre mice (*n* = 14 per group) after intraperitoneal injection with 100 PFU of EMCV virus. (**F**) RT-qPCR of mRNA expression for EMCV replicase in heart and brain tissues from *Mavs*^−/−^ *Dhx9*^f/f^ and *Mavs*^−/−^ *Dhx9*^f/f^ LysM-Cre mice 2 days after intraperitoneal injection, shown as means ± SEM of *n* ≥ 10. Statistical analysis in (E) was performed by Kaplan-Meier analysis and, in (F), was performed by unpaired Student’s *t* test; *P < 0.05.

As a multifunctional protein, DHX9 is reportedly located in both the nucleus and cytoplasm in different cell types (*20*, *28*), which participate in nearly every cellular pathway involving RNA (*29*). We first validated the subcellular localization of DHX9 in RNA viral infections. Fluorescence microscopy showed that DHX9 was mainly located in the nucleus in VSV-GFP–infected Hela cells (GFP positive). There was no notable change in DHX9 localization between infected and uninfected cells (Fig. 3A). Consistent with this result, the subcellular localization of DHX9 was mainly restricted to the nucleus in MNV-infected BMDMs (dsRNA positive) (Fig. 3B). We also performed subcellular fractionation separation experiments on HeLa cells stimulated with poly(I:C) or VSV, followed by Western blot analysis, and further confirmed the nucleus distribution of DHX9 in a steady state or upon viral infection (Fig. 3, C and D). Therefore, we suspect that DHX9 also plays an essential anti-RNA viral role in the nucleus. However, previous studies have demonstrated that DHX9 acts as a cytosolic dsRNA sensor in myeloid cells and pairs with MAVS to activate downstream innate immune responses (*10*). We further crossed *Dhx9*^f/f^ LysM-Cre mice with *Mavs*^−/−^ mice to explore the potential antiviral function of DHX9 in addition to RNA sensing. *Mavs*^−/−^ *Dhx9*^f/f^ LysM-Cre and their littermates *Mavs*^−/−^ *Dhx9*^f/f^ mice were infected with EMCV by intraperitoneal injection. *Mavs*^−/−^ *Dhx9*^f/f^ LysM-Cre mice showed higher sensitivity to EMCV viral infections in terms of survival rate (Fig. 3E) and higher viral loads in the heart and brain than in *Mavs*^−/−^ *Dhx9*^f/f^ mice (Fig. 3F). This further suggests that DHX9 has anti-RNA viral functions other than cytoplasmic RNA sensors. In conclusion, DHX9 is primarily concentrated in the nucleus under infected conditions, and resistance to RNA virus infection is partially independent of RNA sensing.

### DHX9 deficiency impairs IFN-induced antiviral response

**Fig. 4.**
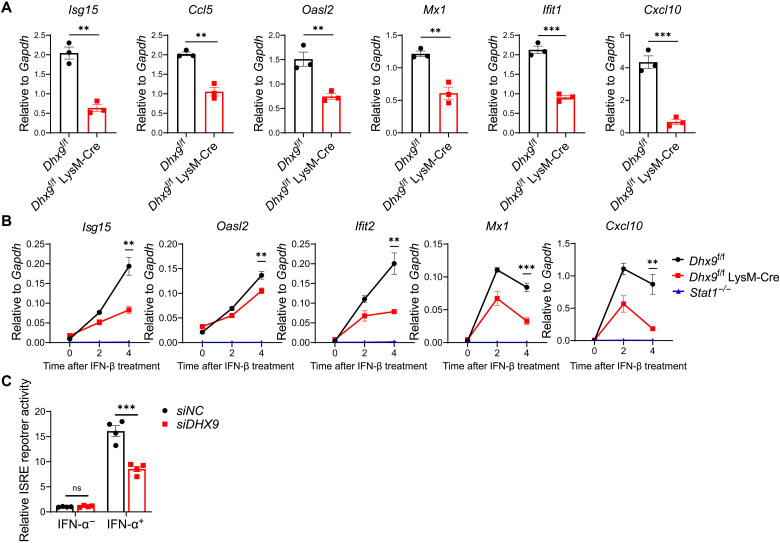
DHX9 deficiency impairs type I IFN–induced antiviral response. (**A**) RT-qPCR analysis of ISGs (*Isg15*, *Ccl5*, *Oasl2*, *Mx1*, *Ifit1*, and *Cxcl10*) expression in peritoneal macrophages from *Dhx9*^f/f^ and *Dhx9*^f/f^ LysM-Cre mice stimulated with IFN-β for 4 hours. Data represent means ± SEM of three independent experiments. (**B**) RT-qPCR analysis of the expression of *Isg15*, *Oasl2*, *Ifit2*, *Mx1*, and *Cxcl10* from *Dhx9*^f/f^, *Dhx9*^f/f^ LysM-Cre, and *Stat1*^−/-^BMDMs stimulated with IFN-β at the time points indicated (0, 2, and 4 hours). Data represent means ± SEM of three independent experiments. (**C**) HEK293T cells were transfected with 50 nM siNC or siDHX9 siRNA. Twenty-four hours after transfection, ISRE luciferase reporter plasmids (100 ng) and *Renilla* luciferase reporter plasmids (10 ng) were cotransfected and incubated for 24 hours. Subsequently, cells were stimulated with IFN-α, and luciferase activity was detected 24 hours after IFN-α stimulation. Data represent means ± SEM of four independent experiments. Statistical analysis was performed by two-tailed unpaired Student’s *t* test;**P < 0.01, and ***P < 0.001.

In addition to sensing dsRNA and CpG DNA in the cytoplasm, DHX9 has been extensively characterized as a transcriptional regulator, which is consistent with its mostly nuclear localization (*18*). In the RHV and EMCV infection models, we observed a significant difference in ISG production in DHX9-deficient cells. Therefore, we hypothesized that DHX9 could directly regulate ISG expression downstream of IFN. We first performed an RT-qPCR analysis of ISG expression in peritoneal macrophages from *Dhx9*^f/f^ and *Dhx9*^f/f^ LysM-Cre mice stimulated with IFN-β. Compared with *Dhx9*^f/f^ peritoneal macrophages, the expression of IFN-β downstream genes *Isg15*, *Ccl5*, *Oasl2*, *Mx1*, *Ifit1*, and *Cxcl10* significantly decreased in *Dhx9*^f/f^ LysM-Cre after IFN-β treatment (Fig. 4A). We observed a similar decreasing trend in the expression levels of ISGs (*Isg15*, *Ccl5*, *Oasl2*, *Mx1*, *Ifit1*, and *Cxcl10*) in *Dhx9*^f/f^ LysM-Cre upon IFN-β stimulation at different time points (0, 2, and 4 hours), compared to *Dhx9*^f/f^ BMDMs (Fig. 4B). Consistently, we also found that the depletion of DHX9 significantly impaired the ISG expression in primary hepatocytes upon IFN-β stimulation (fig. S5). Previous studies have demonstrated that DHX9 is recruited to promyelocytic leukemia nuclear bodies (PML-NBs) in response to IFN-α stimulation and could be involved in the transcriptional regulation of some ISGs attached to PML-NBs (*30*). Evidence exists that PML is associate with transcription factor complexes that control IFN and ISG expression (*31*–*33*). To test whether DHX9 facilitates STAT1-mediated transcription in a manner that involves PML, we constructed PML knockout Hela cells (fig. S6, A and B) and then tested whether the silence of DHX9 by small interfering RNA (siRNA) still affected ISG expression in PML-KO cells in response to IFN-α stimulation. Our results showed that PML deficiency did not affect DHX9-mediated induction of ISG transcription (fig. S6, C and D), indicative of a PML-independent transcriptional mechanism. We next examined the effects of DHX9 in IFN-α–induced IFN-stimulated response element (ISRE) luciferase reporter activities. Human embryonic kidney (HEK) 293T cells were transfected with negative control siRNA (siNC) or siDHX9 siRNA. Twenty-four hours after transfection, ISRE luciferase reporter plasmids and *Renilla* luciferase reporter plasmids were cotransfected and stimulated with IFN-α. ISRE reporter activities significantly decreased in DHX9-silenced cells upon IFN-α induction (Fig. 4C). Together, these findings suggest that DHX9 promotes type I IFN–induced ISGs.

### DHX9 binds to STAT1 in response to IFN stimulation

**Fig. 5.**
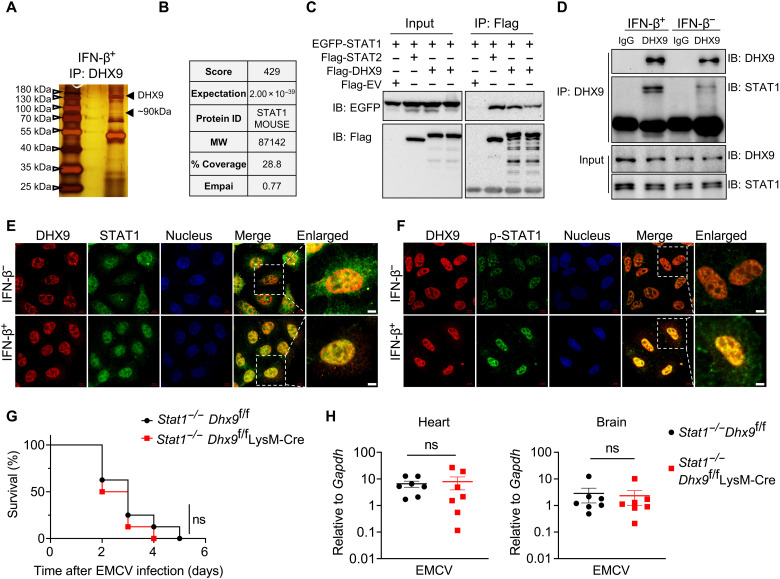
DHX9 binds to STAT1 in response to type I IFN stimulation. (**A**) Silver-stained gel image for specific proteins was immunoprecipitated from whole-cell lysates of BMDMs stimulated with IFN-β (250 U/ml) for 2 hours using an antibody directed against DHX9. (**B**) Gel pieces containing regions of interest were analyzed by LC-MS/MS to identify proteins coimmunoprecipitated with DHX9. STAT1 was specifically identified. MW, molecular weight. (**C**) Flag-tagged DHX9 or Flag-tagged STAT2 was transfected with the indicated GFP-tagged STAT1 into HEK293T cells. After 36 hours, cell lysates were collected, then immunoprecipitated, and blotted as indicated. (**D**) BMDMs from *Dhx9*^f/f^ and *Dhx9*^f/f^ LysM-Cre mice were stimulated with IFN-β (250 U/ml) for 2 hours, and the cells were collected, then immunoprecipitated, and blotted as indicated. IB, immunoblotting. (**E**) Representative confocal microscopy images of Hela cells that were stimulated with IFN-β (1000 U/ml); the staining of DHX9 and STAT1 is shown as indicated. Scale bars, 5 μm. (**F**) Representative confocal microscopy images of Hela cells that were stimulated with IFN-β (1000 U/ml); the staining of DHX9 and p-STAT1 is shown as indicated. Scale bars, 5 μm. (**G**) Survival rates of age- and sex-matched *Stat1*^−/−^ *Dhx9*^f/f^ and *Stat1*^−/−^ *Dhx9*^f/f^ LysM-Cre mice (*n* = 8 per group) after intraperitoneal injection with 50 PFU of EMCV virus. (**H**) RT-qPCR of mRNA expression for EMCV replicase in heart and brain tissues from age- and sex-matched *Stat1*^−/−^ *Dhx9*^f/f^ and *Stat1*^−/−^ *Dhx9*^f/f^ LysM-Cre mice 1 day after intraperitoneal injection, shown as means ± SEM of *n* = 7. Statistical analysis in (G) was performed by Kaplan-Meier analysis and, in (H), by unpaired Student’s *t* test; ns, not significant (*P* > 0.05).

To explore the mechanism by which DHX9 regulates ISG production, we performed immunoprecipitation (IP) coupled with mass spectrometry analysis of the DHX9-interacting proteins. First, mature BMDMs were stimulated with IFN-β for 2 hours, and cell lysates were coincubated with DHX9 antibody–conjugated agarose beads, with isotype control immunoglobulin G (IgG)–conjugated agarose beads as the negative control. Specific bands from DHX9 antibody–IP samples were then subjected to mass spectrometry (Fig. 5A). In particular, we identified that STAT1, a key protein regulating ISGs, could interact with DHX9 (Fig. 5B). The interaction between STAT1 and DHX9 was validated by coimmunoprecipitation (co-IP) in HEK293T cells, and the interaction between STAT1 and STAT2 was used as a positive control. As expected, STAT2 strongly interacts with STAT1, and DHX9 was also found to interact with STAT1 at a comparable level (Fig. 5C). Furthermore, we examined the endogenous interaction between DHX9 and STAT1 in BMDMs using the DHX9 antibody for IP. The results showed a weak interaction between DHX9 and STAT1 in a steady state while a strong inducible interaction upon IFN-β stimulation (Fig. 5D).

Next, we examined the subcellular localization of DHX9 and STAT1 in HeLa cells stimulated with IFN-β by immunofluorescence assay (IFA). In response to IFN-β stimuli, STAT1 is translocated to the nucleus, where most DHX9 is located, and nucleic STAT1 and phosphorylated STAT1 (p-STAT1) colocalized with DHX9 well in the nucleus (Fig. 5, E and F). Thus, DHX9 may regulate ISG production by directly interacting with p-STAT1. Since type II IFN (IFN-γ) can also induce the phosphorylation and nuclear translocation of STAT1 to transcribe ISGs, we also detected the expression of ISGs in *Dhx9*^f/f^ and *Dhx9*^f/f^ LysM-Cre BMDMs under IFN-γ stimulation. The results showed that the expression of ISGs downstream of IFN-γ (*Ciita*, *Icam1*, and *Stat1*) was also significantly decreased in the absence of DHX9 (fig. S7).

Furthermore, we crossed *Dhx9*^f/f^ LysM-Cre mice with *Stat1*^−/−^ mice to explore the potential antiviral function of DHX9 mediated by STAT1. *Stat1*^−/−^ *Dhx9*^f/f^ LysM-Cre and littermates *Stat1*^−/−^ *Dhx9*^f/f^ mice were infected with EMCV by intraperitoneal injection. Notably, there were no significant differences in either survival curves or virus titers in organs between these two mouse strains (Fig. 5, G and H). These results showed that DHX9 functionally interacts with STAT1 in the nucleus to mediate antiviral response.

### DHX9 promotes STAT1-transcribed ISG expression

**Fig. 6.**
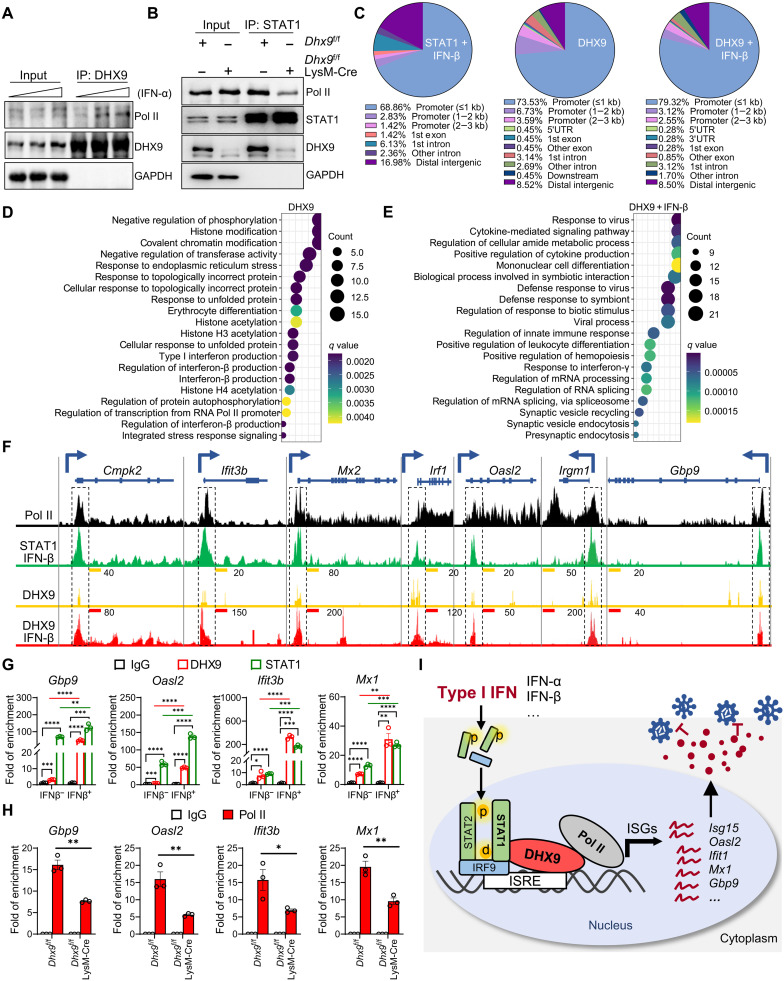
DHX9 promotes STAT1-transcribed ISGs. (**A**) HEK293T cells were stimulated with different concentrations of IFN-α (100, 1000, and 10,000 U/ml), followed by IP-DHX9 detection of Pol II protein expression, and blotted as indicated. (**B**) BMDMs from *Dhx9*^f/f^ and *Dhx9*^f/f^ LysM-Cre mice were stimulated with IFN-β (250 U/ml) for 2 hours, followed by IP of STAT1 and detection of indicated protein by Western blotting. (**C**) Cut&Tag ChIP analysis BMDMs from wild-type mice stimulated with IFN-β (250 U/ml) or phosphate-buffered saline vehicle control for 2 hours. Genomic annotation of DHX9 or STAT1-binding sites from ChIP-Seq data was showed. 5′UTR, 5′ untranslated region. (**D**) Gene ontology analysis the corresponding genes of DHX9-enriched promoter peaks (under steady state) in (C). (**E**) Gene ontology analysis the corresponding genes of DHX9-enriched promoter peaks (upon IFN-β stimulation) in (C). (**F**) UCSC genome browser tracks showing ChIP-Seq of Pol II–, STAT1 (stimulated by INF-β)–, and DHX9 (with or without INF-β stimulation)–bound promoter regions of ISGs in BMDMs. The Pol II ChIP-Seq raw data were downloaded from gene expression omnibus (GEO) (accession no. GSE106706). (**G**) ChIP-qPCR validation of DHX9 or STAT1-binding sites in ISGs (*Gbp9*, *Oasl2*, *Ifit3b*, and *Mx1*) promoter DNA with or without IFN-β stimulation; data represent means ± SEM of three independent experiments. (**H**) ChIP-qPCR analysis of Pol II–bound sites in ISG (*Gbp9*, *Oasl2*, *Ifit3b*, and *Mx1*) promoters with or without IFN-β stimulation. Data represent means ± SEM of three independent experiments. (**I**) A proposed model of nucleic DHX9 cooperates with STAT1 to transcribe ISGs. Statistical analysis in (G) and (H) was performed by two-tailed unpaired Student’s *t* test. *P < 0.05, **P < 0.01, ***P < 0.001, and ****P < 0.0001.

The activation of STAT1 mainly depends on phosphorylation at the Tyr^701^ site (pY701-STAT1) catalyzed by kinase JAK1, which is crucial for type I IFN (IFN-α/β)–induced cellular antiviral signaling. Therefore, we tested whether DHX9 affects STAT1 phosphorylation at Tyr^701^. We found that pY701-STAT1 levels were comparable in BMDMs from *Dhx9*^f/f^ LysM-Cre and *Dhx9*^f/f^ mice after IFN-β stimulation (fig. S8). DHX9 has been extensively characterized as a transcriptional regulator (*18*) and is a bridging factor between the transcriptional coactivator adenosine 3′,5′-monophosphate response element–binding protein (CREB)–binding protein (CBP)/p300 and RNA polymerase II (RNA Pol II) (*34*). In vitro experiments indicating that DHX9 can unwind aberrant structures (RNA forks, R-loops, and RNA-based tetraplexes) during stalled transcription also strongly suggested the role of DHX9 in transcription (*35*). Hence, we hypothesized that DHX9 could bridge the interaction between STAT1 and RNA Pol II to facilitate ISG transcription. We first validated the potential binding between DHX9 and Pol II. We found an increased interaction between DHX9 and Pol II upon IFN-α stimulation (Fig. 6A). Next, we performed co-IP between endogenous Pol II and STAT1 using STAT1 antibody in *Dhx9*^f/f^ and *Dhx9*^f/f^ LysM-Cre BMDMs stimulated with IFN-β. The results showed that the interaction between endogenous STAT1 and Pol II is much reduced in the absence of DHX9 (Fig. 6B). Thus, DHX9 is involved in the formation of the transcription complex that initiates the ISG transcription.

To explore the potential role of DHX9 in transcription along with STAT1, we performed CUT&Tag-seq, a new generation of chromatin IP sequencing (ChIP-seq) with high repeatability and fine signal-to-noise ratio, with BMDMs treated with IFN-β or negative control for 2 hours. Subsequently, cells were fixed, and a CUT&Tag-seq assay was performed using DHX9 or STAT1-specific antibodies. The specifically bound DNA was then sequenced. We found that most of the binging peaks of DHX9 or STAT1 are within the promoter region: 83.86% (≤1 kb: 73.54%, 1 to 2 kb: 6.73%, and 2 to 3 kb: 3.59%) of DHX9 binding peaks (not stimulated), 84.99% (≤1 kb: 79.32%, 1 to 2 kb: 3.12%, and 2 to 3 kb: 2.55%) of DHX9 binding peaks (stimulated by IFN-β), and 71.30% (≤1 kb: 68.87%, 1 to 2 kb: 2.83%, and 2 to 3 kb: 1.42%) of STAT1 binding peaks (stimulated by IFN-β, as a control) in BMDMs were in the promoter regions relative to gene annotations (Fig. 6C). Since promoter regions accounted for only 4.41% of the reference genome (*36*), DHX9 preferentially binds to the promoter region. Moreover, by gene ontology pathway analysis, we found the IFN-β–induced change of DHX9-bound promoters: In steady state, DHX9 bound to promoters of genes that are involved in histone and chromatin modification and regulation (such as regulation of phosphorylation, histone modification, and covalent chromatin modification), regulation of transcription from RNA polymerase II promoter, and regulation of type I IFN pathways (Fig. 6D). Notably, upon IFN stimulation (DHX9 + IFN-β group), ISG production was the most enriched pathway of DHX9 bounds to promoters of genes (such as response to virus, positive regulation of cytokine production, and response to IFN-γ) (Fig. 6E). Thus, the specific binding of DHX9 to ISG promoters was significantly increased after IFN stimulation. Heatmap also showed that STAT1 and DHX9 binding peaks were greatly enriched at transcription start sites in BMDMs (fig. S9, A and B). Consistently, our ChIP-seq showed that both DHX9 and STAT1 were bound to the promoters of ISGs (Fig. 6F and fig. S9C). Notably, the binding of DHX9 to promoters of ISGs was significantly increased after INF-β stimulation, including *Cmpk2*, *Ifit3b*, *Mx2*, *Irf1*, *Oasl2*, *Irgm1*, *Gbp9*, *Mx1*, *ifi204*, *Oasl1*, *Stat1*, *Usp18*, and, *Ifih1* (Fig. 6F and fig. S9C). In addition, our ChiP-seq motif enrichment analysis showed that the promoter and enhancer regions of ISGs, ISREs, were specificity enriched in the DHX9 binding peaks after IFN-β stimulation (fig. S10, A and B). We next validated the ChIP-seq data by ChIP-qPCR assay and detected significantly higher DHX9 and STAT1 binding in the ISGs (*Gbp9*, *Oasl2*, *Ifit3b*, and *Mx1*) promoter region in IFN-β–treated BMDMs (Fig. 6G), confirming the recruitment of DHX9 and STAT1 to the ISG promoters during IFN-β.

We reanalyzed the previously published Pol II ChIP-seq data (*37*); the binding peaks overlap well with the DHX9 and STAT1 peaks (Fig. 6F and fig. S9C), further supporting STAT1/DHX9/Pol II may work together to transcribe ISGs. To further confirm that the recruitment of Pol II to the promoters of ISGs is dependent on DHX9, we performed a ChIP assay using Pol II antibody in *Dhx9*^f/f^ and *Dhx9*^f/f^ LysM-Cre BMDMs stimulated with IFN-β. The results showed that the recruitment of Pol II to the promoters of ISGs was significantly reduced in the absence of DHX9 (Fig. 6H). Therefore, our data suggest that DHX9 acts as a transcriptional coactivator by recruiting Pol II to promote STAT1-mediated transcription of ISGs (Fig. 6I).

## DISCUSSION

The DExD/H-box helicases are widely involved in physiological processes such as RNA metabolism (*38*). They are also involved in host antiviral immunity (*19*). Because of the detrimental function of DExD/H-box helicases in cellular processes and the lethality of these helicase-deficient mice, few in vivo experiments have been performed to characterize the antiviral role of RNA helicase. DHX9, a key player in regulating RNA splicing and translation, has been shown in vitro to either promote antiviral immune response (*10*, *21*) or assist the viral replication in cells (*19*). Hence, in vivo experiments are urgently needed to determine the role of DHX9 in innate immune response toward viruses. In this study, we generated myeloid-specific and hepatocyte-specific DHX9 knockout mice (*Dhx9*^f/f^ LysM-Cre and *Dhx9*^f/f^ Alb-Cre) and tested the in vivo role of DHX9 in anti-RNA virus immune responses in macrophages and hepatocytes.

Previously, DExD/H-box helicase family proteins were thought to play an antiviral role in the cytoplasm, such as DDX3 (*39*), DHX15 (*40*), DHX33 (*41*), and DHX9 (*10*), by sensing the viral RNA or cooperating with MAVS to activate downstream antiviral signals. However, our results showed that the antiviral effect of DHX9 was likely independent of its cytoplasmic RNA sensor function. Recently, there have been cases showing that RNA helicases can also modulate innate immune responses in the nucleus. For instance, DDX46 recruits ALKBH5, an “eraser” of the RNA modification N6-methyladenosine (m6A), via DDX46 DEAD helicase domain to demethylate m6A-modified antiviral transcripts after viral infection (*42*); DDX3X is recruited to the enhanceosome-binding site on the IFN-β promoter that is necessary for type I IFN induction (*43*). The newly identified nucleic role of DHX9 in cooperating with STAT1 to transcribe the ISG mRNA further confirmed the nucleic roles of RNA helicases in antiviral responses. Notably, a recent study also showed that DHX9 plays an integral role in maintaining the antiviral function of CD8^+^ T cells (*44*). There were no developmental abnormalities for CD8^+^ T cells in the thymi and spleens after DHX9 deletion, while loss of DHX9 impairs the accumulation of CD8^+^ effector and memory T cells upon viral infection. However, our results showed that the loss of DHX9 in macrophages did not affect the proportion of mature macrophages either in steady state or 2 days after EMCV infection. The difference in the survival of DHX9-deficient cells after infection might be due to different types of viruses, different duration, or different cell types.

We provided detailed evidence in this work showing how DHX9 participates in ISG transcription. Upon IFN stimulation, DHX9 directly binds to STAT1 in the nucleus; both DHX9 and STAT1 bind to the same promoter region of ISGs. Previous studies have shown that DHX9 can simultaneously bind transcription factors and RNA Pol II, acting as a bridging factor. For instance, DHX9 links RNA Pol II to breast cancer type 1 susceptibility protein (BRCA1) to initiate the transcription, in addition to its roles in DNA replication and DNA repair (*45*). DHX9 also serves as an adaptor to link nuclear β-actin with RNA Pol II, which enhances transcription from the actin-dependent colony-stimulating factor-1 (CSF-1) promoter (*46*). DHX9 directly links p65 and RNA Pol II and enhances NF-κB–dependent transcriptional activation (*22*, *47*). Notably, DHX9 binds directly to both the transcriptional coactivator CBP/p300 and to RNA Pol II, recruiting the latter to the CBP/p300 complexes at the promoter region (*34*). As STAT1 can directly use CBP/p300 as a transcriptional coactivator (*48*), it is possible that DHX9, STAT1, CBP/p300, and Pol II form a transactivation complex in response to IFN stimulation. Multiple domains of DHX9 might be involved in the STAT1-mediated transcription of ISGs. First, the C-terminal oligosaccharide-binding fold domain contains a nuclear localization signal, which is required for DHX9’s localization and function in the nucleus (*29*). Second, previous studies have confirmed that the minimal transactivation domain (MTAD) of DHX9 is critical for CREB-dependent transcription and interaction with Pol II (*17*); we therefore speculate that MTAD might be also required for DHX9 to recruit Pol II to participate in STAT1-mediated transcription of ISGs. Third, the adenosine triphosphate/helicase core domain (HCD), a key region of DHX9 adenosine triphosphatase/helicase activity, has been shown before to promote CBP-mediated and NF-κB–mediated transcription (*22*, *34*). Therefore, The HCD of DHX9 may be also involved in the STAT1-mediated transcription of ISGs. It is worthwhile to further investigate whether these domains, as well as the other domains, such as dsRNA-binding domain and helicase-associated domain 2, are involved in STAT1-mediated transcription of ISGs.

Upon type I IFN stimulation, the ISGF3 complex (consists of a STAT1-STAT2 heterodimer and the IRF9 protein) is the predominant transcriptional factor binding to ISREs (consensus sequence TTTCNNTTTC) within the promoter and enhancer region of ISG. Similar to other transcription factors, ISGF3 transcription involves several coactivator and cofactor proteins that mediate interactions with some transcription machines and chromatin modification enzymes (*49*). For instance, histone acetyltransferase (HAT) activity transforms chromatin into a more relaxed structure, STAT2 binding with HAT family members CBP/p300 and GCN5 enhances the transcriptional activity of ISGs (*50*, *51*), and the chromatin-remodeling factor BRG1 interacts with STAT2 to promote the selective transcription of a subset of ISGs by regulating the cis-acting and trans-acting elements of a subset of the IFN-α-regulated target genes (*52*). The current study shows that DHX9 acts as a coactivator of ISGs and also provides a reasonable model for how DHX9 interacts with STAT1 to recruit Pol II to the promoters and enhancers of ISGs. Furthermore, ISGs are also reported to restrict the infection of DNA viruses (*53*). Since we found that DHX9 bridge the STAT1 and Poll II to transcribe ISGs, we speculate that DHX9 is also important for the anti-DNA virus immune response. A previous study showed that DHX9 inhibits the replication of Murine herpesvirus 68 and the Herpes simplex virus 1, two well-known dsDNA viruses (*22*), which is consistent with our observation of the DHX9 function in anti-RNA virus and ISG transcription.

In summary, we confirmed the function of DHX9 in anti-RNA viral immune response using DHX9 conditional knockout mice. Our study provides in vitro and in vivo evidence to demonstrate that DHX9 acts as a transcriptional coactivator in the nucleus in addition to its traditional dsRNA sensing role in the cytoplasm. Mechanistically, DHX9 interacts with STAT1 in the nucleus to recruit Pol II to participate in STAT1-mediated transcription and facilitate ISG expression to antagonize RNA viruses. This study strengthens our understanding of the antiviral function of DHX9 and the ISG transcriptional machinery.

## MATERIALS AND METHODS

### Mice

*Dhx9*-floxed mice were generated using the CRISPR-Cas9–based genome-editing system (*54*). The upstream lox site was inserted into the intron region between the first and second exons, and the downstream lox site was in the intron region downstream of the 28th exon. Genotyping of *Dhx9*-floxed mice was performed using universal primers 5′-TTTTGACCAG AGTGTGCAGA A-3′ and 5′-CCTACAATGG TTATTGTTGT AGACTGA-3′, with amplicons of a 249-bp product from the wild-type allele and a 283-bp product from the targeted allele. In addition, the *Dhx9*^f/f^ mice were crossed with LysM-Cre or Alb-Cre transgenic mice (The Jackson Laboratory) that express Cre recombinase in myeloid cells and hepatocytes to generate myeloid-specific and hepatocyte-specific Dhx9 knockout mice. *Mavs*^−/−^ and *Stat1*^−/−^ mice were as described in our previous report (*55*). All animal experiments used 6- to 8-week-old, age- and sex-matched mice in C57BL/6 background. Mice were maintained under a strict 12-hour light/12-hour dark cycle, with lights on from 8 a.m. to 8 p.m. All animal experiments were approved by the Ethics Committee of the University of Science and Technology of China.

### Cells and viruses

HEK293T cells, HeLa cells, Vero cells, and RAW 264.7 cells were maintained in Dulbecco’s modified Eagle’s medium (DMEM; Life Technologies) containing antibiotics (Life Technologies) and 10% fetal bovine serum (Life Technologies). To generate BMDMs, we isolated and cultured mouse tibia and femoral bone marrow cells for 7 days in DMEM complemented with 10% fetal bovine serum and supplemented with 30% of the L929 cell supernatant as a source of macrophage colony-stimulating factor.

VSV Indiana strain and VSV-GFP were propagated and amplified by infecting a monolayer of Vero cells. IAV (A/PR/8/34) was amplified in 10-day-old specific pathogen–free embryonated eggs. VSV and IAV were maintained in our laboratory. MNV1 and MNV3 (provided by S. Zhu, Zhejiang University) were amplified by infecting a monolayer of RAW 264.7 cells. EMCV was described in our previous report (*12*). The viral stocks of RHV-1 were prepared according to previous studies (*56*, *57*). Briefly, the complete genome of RHV-rn1 (accession no. KX905133) was synthesized, linearly digested, and served as a template for in vitro transcription. *Mavs*^−/−^ C57/B6 mice were then intrahepatically injected with 5 μg of purified RNA by tail vein injection. The serum from infected mice was obtained 3 days after injection and continually passaged through the *Mavs*^−/−^ mice three times to obtain higher titer viral stocks. Titers of RHV-1 virus stocks were determined by RT-qPCR. The viruses were stored at −80°C.

### Reagents

The poly(I:C) (both HMW and LMW) were purchased from InvivoGen. Recombinant mouse IFN-β was purchased from Sino Biological (50708-MCCH), and recombinant human IFN-α2b injections were from Kaiyin (S20030032). Recombinant mouse IFN-γ was purchased from Sino Biological (50709-MNAH). Lipofectamine 3000 and Lipofectamine RNAiMAX were purchased from Invitrogen. Protein A/G agarose, used for IP, was purchased from Pierce (20423). Antibodies specific for STAT1 (9172, 1:1000 for Western blotting; 14994, 1:200 for IP) and p-STAT1 (9167, 1:1000) were obtained from Cell Signaling Technology. Antibodies to DHX9 were obtained from Abcam (ab26271, 1:2000) and Proteintech (67153, 1:1000). RNA pol II antibodies were purchased from Active Motif (91151, 1:500) and Abcam (ab300575). Antibody to PML was obtained from Proteintech (21041). Flag-M2 agarose beads (A2220) were obtained from Sigma-Aldrich. siRNA targeting DHX9 or nontarget control siRNA (siNC) were purchased from GenePharma (Shanghai, China). siRNA sequences of DHX9 are as follows: DHX9-homo-598 (GAGCCAACUU GAAGGAUUAT T and UAAUCCUUCA AGUUGGCUCT T) and DHX9-homo-2944 (CCUGGGAUGA UGCUAGAAUT T and AUUCUAGCAU CAUCCCAGGT T).

### Immunoblotting and IP

Cells were harvested using lysis buffer containing 50 mM tris-HCl (pH 7.4), 2 mM EDTA, 150 mM NaCl, 0.5% NP-40, phenylmethylsulfonyl fluoride (50 μg/ml), and complete protease inhibitor mixtures (Sigma-Aldrich). Lysates were cleared by centrifugation, the protein concentrations were measured, and equal amounts of lysates were used for immunoblotting and IP. In the IP assay, specific antibody-conjugated protein A/G agarose beads were added into samples and incubated on a rotor at 4°C overnight. After four washes with lysis buffer, proteins bound to beads were eluted by boiling with the loading buffer for 10 min. Then, the samples were analyzed by SDS–polyacrylamide gel electrophoresis and transferred to polyvinylidene difluoride membranes. Membranes were detected with specific primary antibodies and secondary horseradish peroxidase–conjugated anti-mouse or rabbit antibodies. Immunoreactive bands were visualized using the enhanced chemiluminescence reagent (Millipore).

### Luciferase assay

HEK293T cells were plated on 24-well plates and transfected with 50 nM siNC or siDHX9 siRNA using the Lipofectamine RNAiMAX transfection reagent according to the manufacturer’s instructions. Twenty-four hours after transfection, ISRE luciferase reporter plasmids (100 ng) and *Renilla* luciferase reporter plasmids (10 ng) were cotransfected and incubated at 37°C for 24 hours. Subsequently, cells were stimulated with IFN-α for 24 hours. Luciferase production was assayed using the dual-luciferase reporter assay system (Promega). Polymerase activity was normalized by *Renilla* expression.

### Indirect IFA and confocal microscopy

IFA were done as described (*58*). HeLa cells or BMDMs were grown on coverslips in 24-well plates. After the corresponding experimental treatment, cells were fixed in 4% paraformaldehyde for 30 min at room temperature, subsequently permeabilized, and blocked with phosphate-buffered saline containing 0.5% Triton X-100 and 5% bovine serum albumin for 1 hour at room temperature. For immunostaining, samples were incubated with antibodies against the indicated proteins for 2 hours at room temperature or 4°C overnight, followed by incubation with anti-mouse IgG fluorescein isothiocyanate–conjugated antibody or anti-rabbit IgG Alexa Fluor 594–conjugated antibody for 1 hour. Nuclei were visualized with 4,6-diamidino-2-phenylindole (Invitrogen). All fluorescence images were analyzed via confocal imaging using Zeiss LSM880.

### Nuclear and cytoplasmic fractionation assay

Hela cells were stimulated with poly(I:C) or infected with VSV for 8 hours, and cells were harvested and subjected to nuclear and cytoplasmic protein extraction by the Nuclear and Cytoplasmic Protein Extraction Kit (Beyotime, P0027). The follow-up steps were carried out according to the manufacturer’s instructions. For the cytoplasmic extract, Hela cells were lysed in buffer A and buffer B. Nuclei were then resuspended in nuclear extraction buffer. Each fraction was quantified and proceeded to immunoblotting assay with the following primary antibodies: anti-LAMB1, anti-GAPDH, and anti-DHX9.

### RNA isolation, RT-qPCR, and primers

RNA isolation and RT-qPCR were described previously (*59*). Briefly, total RNAs were isolated from different cell lines or mouse tissues using TRIzol reagent (Invitrogen). The cDNA was synthesized from 500 ng of total RNA using the HiScript III RT SuperMix kit (Vazyme, China) according to the manufacturer’s instructions. After reverse transcription, RT-qPCR was performed on the Bio-Rad CFX384 real-time PCR system in a 10-μl reaction system using SYBR Green qPCR Master Mix (Vazyme, China). *Gapdh* was used as an endogenous control. The mRNA expression levels of the tested genes relative to *Gapdh* were determined using the 2^−ΔΔ*C*t^ method and shown as fold induction. qPCR primers used in this study are provided in the Supplementary Materials (table S1).

### RNA-seq and data analysis

RNA-seq library preparation and sequencing for RHV-infected liver samples and EMCV-infected peritoneal macrophage samples were performed by Berry Genomics on an Illumina 2500 machine. RNA-seq fastq files were processed by Trimmomatic (v0.39) to remove low-quality reads and then aligned to the mouse reference genome (mm10) assembly by STAR (v2.5.3a), followed by quantification with HT-seq (0.11.0). The differentially expressed genes between the experimental groups were identified by edgeR (v3.29.2). Gene ontology enrichment analyses for differentially expressed genes were performed using R package clusterProfiler (v4.0.5).

### Cut&Tag assay and ChIP-qPCR analysis

CUT&Tag assays were performed with the Hyperactive In-Situ ChIP Library Prep Kit for the Illumina kit (TD903, Vazyme) according to the manufacturer’s instructions. Briefly, BMDMs were harvested and transferred into a 1.5-ml low-binding tube. First, prewashed cells were incubated with 10 μl of prewashed concanavalin A–coated magnetic beads. Next, bead-bound cells were incubated with anti-DHX9, anti-STAT1, or normal anti-IgG (Cell Signaling Technology) primary antibodies for 2 hours at room temperature. After washing twice with dig-wash buffer, 50 μl of dig-wash buffer with 0.5 μg of secondary antibody was added and incubated at room temperature for 30 min. After washing twice, 0.58 μl of pG-Tn5 was added to perform tagmentation, and samples were incubated at room temperature for 1 hour. The DNA fragments were then extracted, after which PCR amplification was performed using indexed P5 and P7 primers. All libraries were sequenced by NovaSeq 6000 (Illumina) according to the manufacturer’s instructions. Filtered reads were aligned to the mm10 genome using Bowtie2. PCR duplicates were removed using Picard. Peak calling was done by macs3 (*60*). Motif enrichment analysis was done by HOMER (*61*). An R package ChIPseeker was used for peak annotation (*62*). ChIP-qPCR primers used in this study are provided in the Supplementary Materials (table S2).

### Statistical analysis

All data are presented as means ± SEM. Comparisons between groups were analyzed by unpaired two-tailed Student’s *t* test or one-way/two-way analysis of variance (ANOVA). Statistical analysis was performed using GraphPad Prism 8. *P* < 0.05 was considered to statistically significant (*P < 0.05, **P < 0.01, ***P < 0.001, and ****P < 0.0001; ns, not statistically significant).

## Notes

View/request a protocol for this paper from *Bio-protocol*.
