# Supplementary material for: Nucleic DHX9 cooperates with STAT1 to transcribe interferon-stimulated genes
Source: Sci Adv. 2023 Feb 3;9(5):eadd5005. doi: 10.1126/sciadv.add5005 (PMC9897671; doi:10.1126/sciadv.add5005)
Supplement: Supplementary file 1 — Figs. S1 to S10 Tables S1 and S2 [file sciadv.add5005_sm.pdf]

Science Advances
AAAS

Supplementary Materials for

Nucleic DHX9 cooperates with STAT1 to transcribe interferon-stimulated genes

Xingxing Ren *et al.*

Corresponding author: Shu Zhu, zhushu@ustc.edu.cn; Richard A. Flavell, richard.flavell@yale.edu; Mingsong Li, lims661216@163.com

Sci. Adv. **9**, eadd5005 (2023)
DOI: 10.1126/sciadv.add5005

This PDF file includes:

Figs. S1 to S10
Tables S1 and S2

Fig. S1.

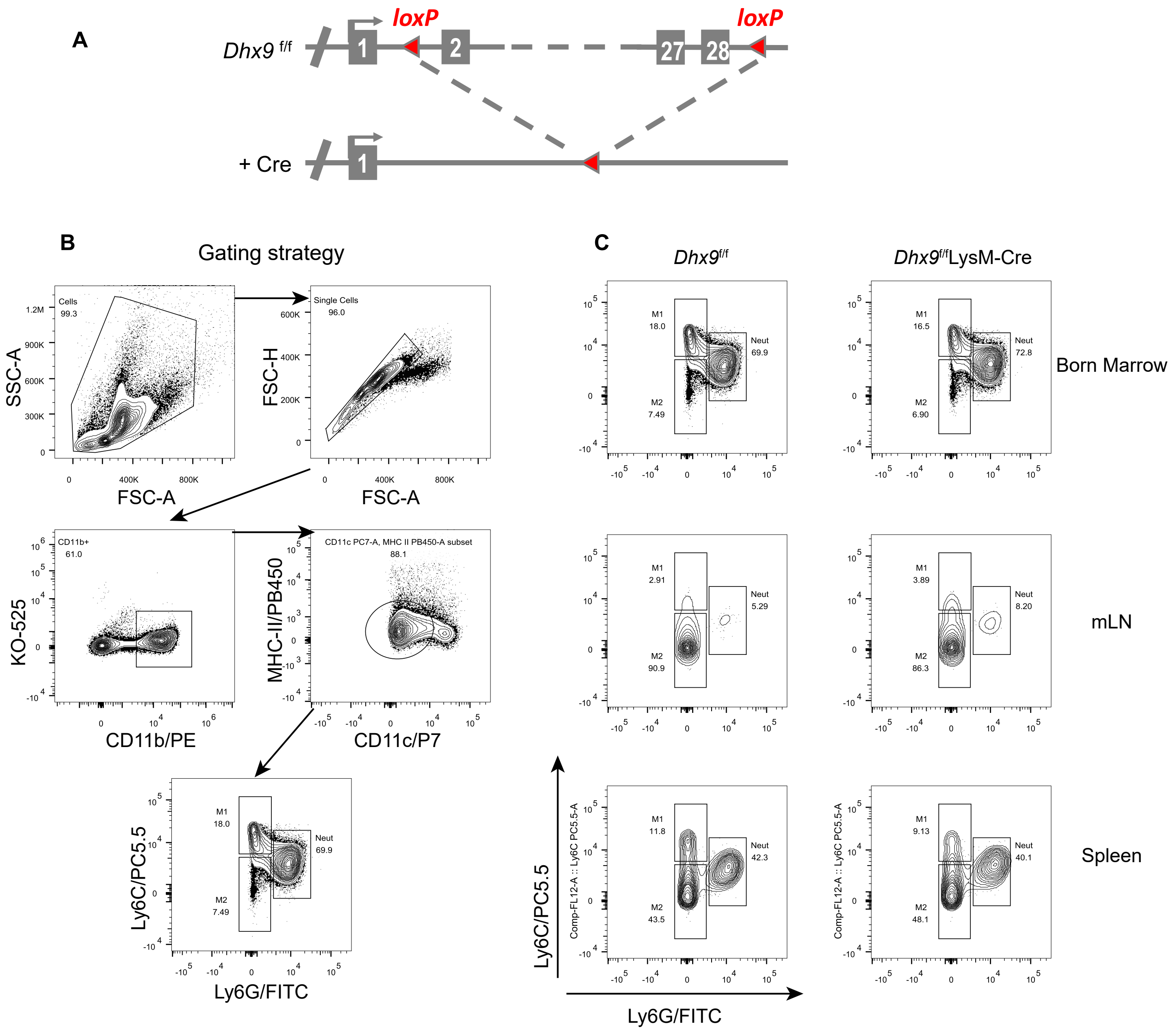

Supplementary Figure 1. The development of myeloid cell is not affected in *Dhx9*$^{f/f}$ LysM-Cre mice

(A) Schematic illustration of the knock-out region of *Dhx9*$^{f/f}$ LysM-Cre mice.

(B) Gating strategy of immune cells in the myeloid cells in bone marrow, spleen, and mLN.

(C) Flow cytometry analysis of the percentages of myeloid cells in bone marrow, spleen, and mLN from *Dhx9*$^{f/f}$ and *Dhx9*$^{f/f}$ LysM-Cre mice. CD11b^{+}/Ly6C^{+}/Ly6G^{-} inflammatory macrophages (M1), CD11b^{+}/Ly6C^{-} /Ly6G^{+} resident macrophages (M2) and neutrophils (CD11b^{+}/Ly6C^{-} /Ly6G^{+}).

Fig. S2.

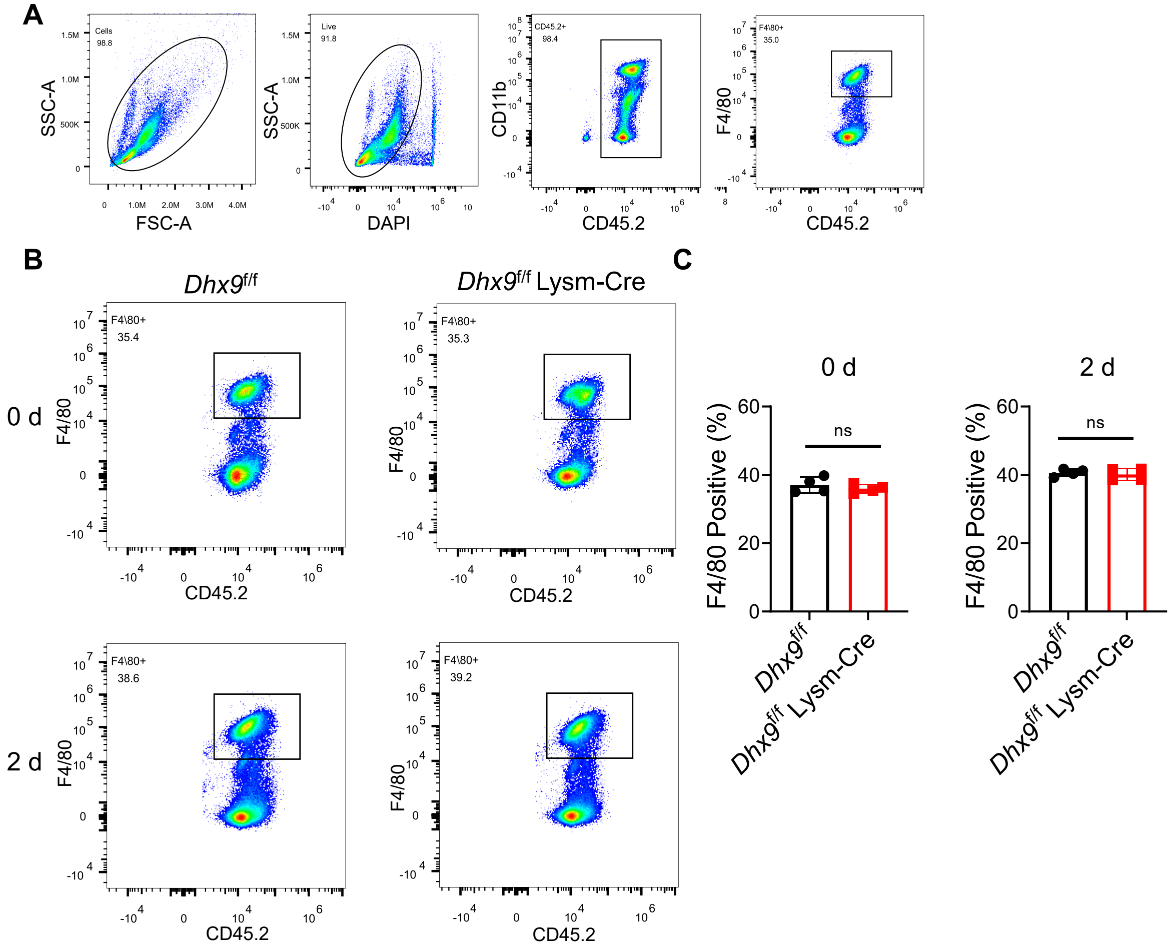

Supplementary Figure 2. The proportion of peritoneal macrophages in Dhx9$^{f/f}$ and Dhx9$^{f/f}$ LysM-Cre mice in steady-state and 2 days post EMCV infection.

(A) Gating strategy of F4/80^{+} macrophages in the peritoneal cells.

(B) Flow cytometry analysis of the percentages of F4/80^{+} macrophages in CD45.2^{+} cells in the peritoneal cells.

(C) Statistics of (B), F4/80^{+} macrophages in CD45.2+ cells (n=4 per group). Data represent means ± SEM and were analyzed by two-tailed, unpaired Student's *t* test. ns, not significant (P > 0.05).

Fig. S3.

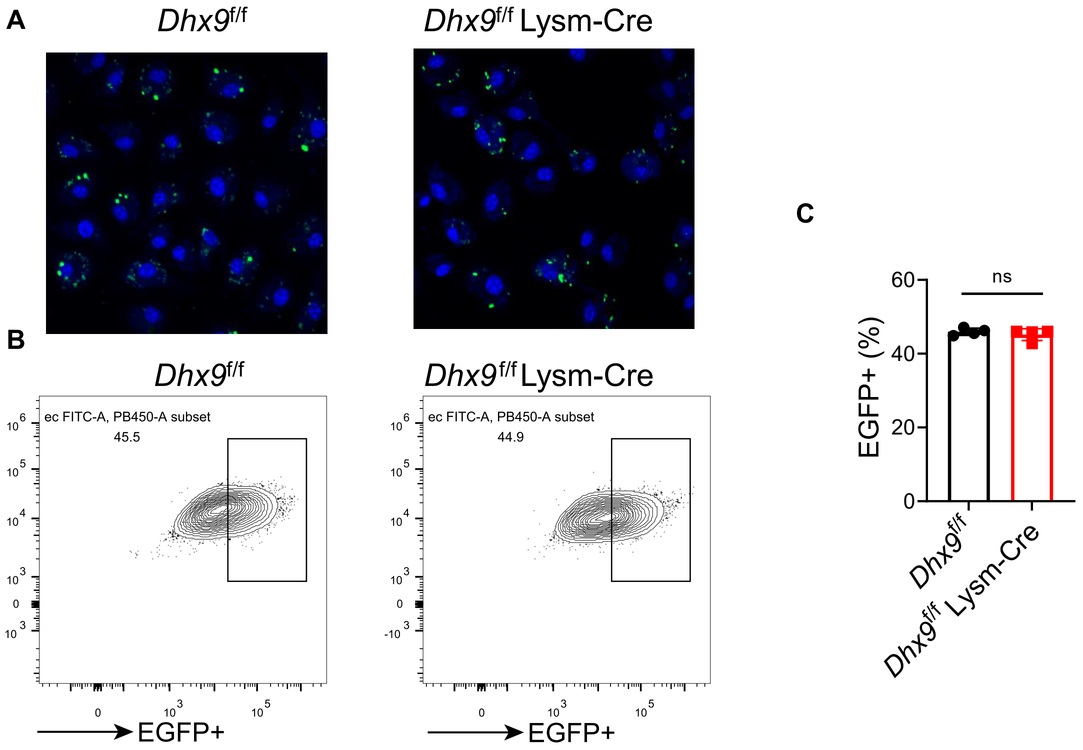

Supplementary Figure 3. Bacterial phagocytic capacity of BMDMs from *Dhx9*$^{f/f}$ and *Dhx9*$^{f/f}$ LysM-Cre mice by bacterial phagocytosis assay using EGFP-E. coli.

(A) Representative confocal-microscopy images of *Dhx9*$^{f/f}$ and *Dhx9*$^{f/f}$ LysM-Cre BMDMs that were added with 20 MOI EGFP-E. coli, and the fluorescence intensity of EGFP was detected 3 h later. Cells nuclei were visualized with DAPI.

(B) Flow cytometry analysis of the percentages of EGFP^{+} macrophages.

(C) Statistics of (B), EGFP^{+} macrophages (n=4 per group). Data represent means ± SEM and were analyzed by two-tailed, unpaired Student's *t* test. ns, not significant ($P > 0.05$).

Fig. S4.

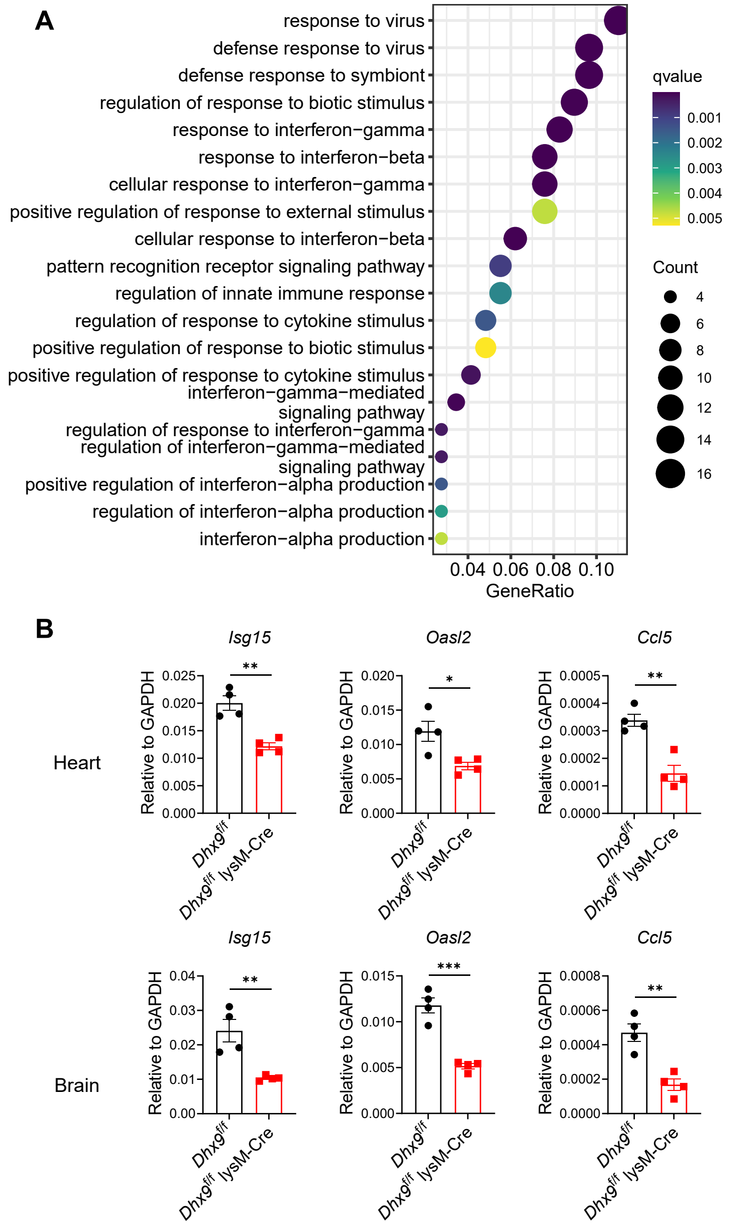

Supplementary Figure 4. DHX9 deficiency dampens the ISG induction

(A) Age and sex-matched *Dhx9*$^{f/f}$ and *Dhx9*$^{f/f}$ LysM-Cre mice were injected with 1000 PFU of EMCV virus by intraperitoneal injection. Sixteen hours post infection, CD11b^{+} peritoneal macrophages were isolated and subjected to RNA-Seq analysis. Gene ontology (GO) analysis of differentially expressed genes in peritoneal macrophage from *Dhx9*$^{f/f}$ LysM-Cre mice compared to *Dhx9*$^{f/f}$ mice according to RNA-Seq data (down-regulated).

(B) RT-qPCR of mRNA expression of *Isg15, Oasl2, and Ccl5* in the heart and brain tissues from *Dhx9*$^{f/f}$ and *Dhx9*$^{f/f}$ LysM-Cre mice 2 days post of EMCV infection. Data represent means ±SEM and were analyzed by two-tailed, unpaired Student's *t* test; *P < 0.05; **P < 0.01; ***P < 0.001.

Fig. S5.

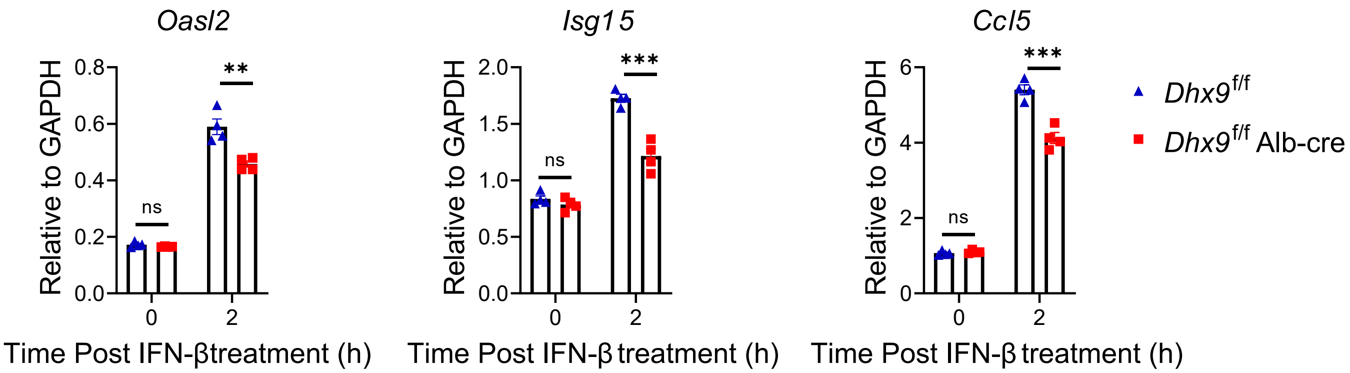

Supplementary Figure 5. DHX9 deficiency impairs type I IFN-induced antiviral response. RT-qPCR analysis the expression of *Oasl2*, *Isg15*, and *Ccl5* from *Dhx9*$^{f/f}$ and *Dhx9*$^{f/f}$ Alb-Cre primary hepatocytes (250 U/mL) for 1 h. Data represent means ± SEM of three independent experiments, and were analyzed by two-tailed, unpaired Student's *t* test; *P < 0.05; **P < 0.01; ***P < 0.001.

Fig. S6.

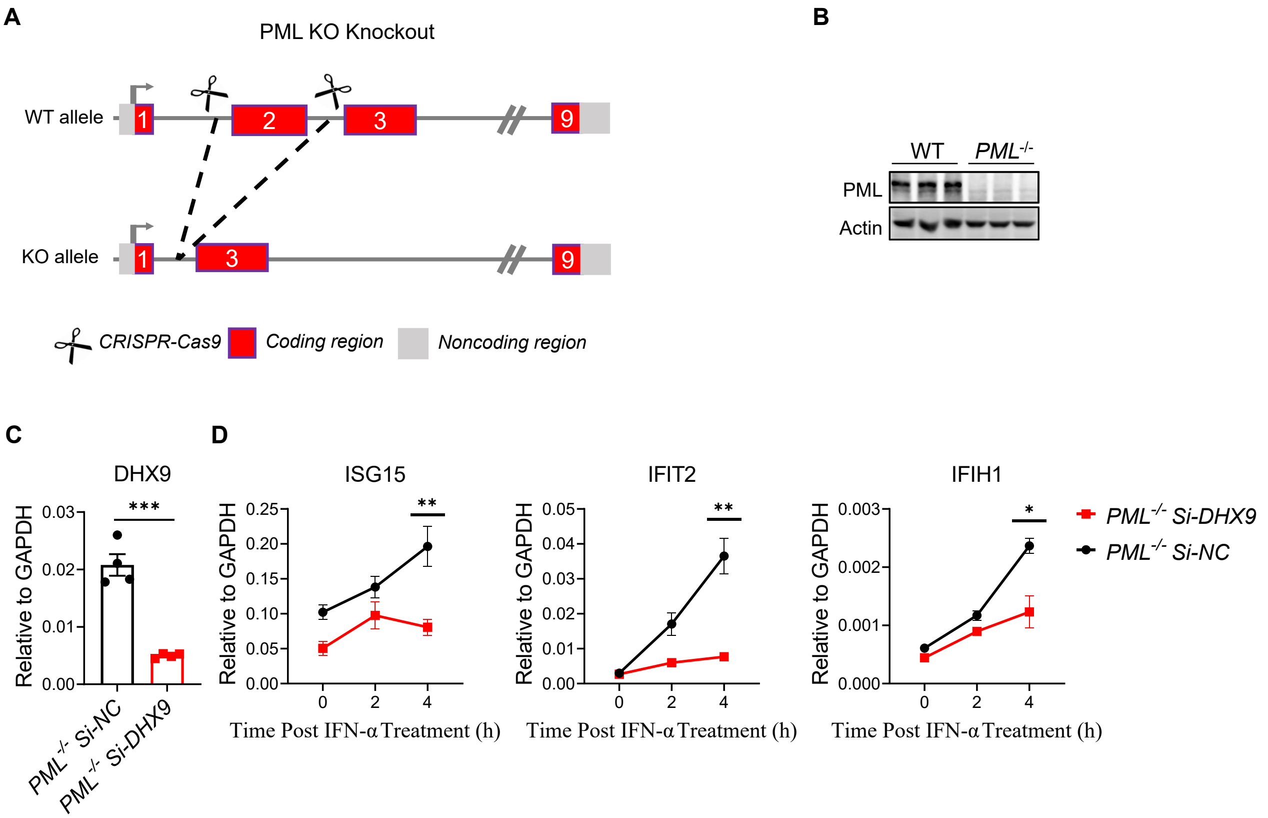

Supplementary Figure 6. PML knockout didn't affect DHX9 mediated induction of ISGs transcription.

(A) Construction of PML gene knockout Hela cell lines by CRISPR-Cas9 system. Information of the deleted fragment in PML-KO cells (799 bp).

(B) Loss of PML protein in PML-KO Hela cells was confirmed by western blotting.

(C) PML-KO cells were transfected with negative control (NC) siRNA or siDHX9 for 36 h, DHX9 knockdown efficiency were evaluated by RT-qPCR analysis;

(D) siNC or siDHX9 PML-KO cells were stimulated with IFN-α (1000 U/mL), 0, 2 h, and 4 h post treatment, the cells were collected for RT-PCR of mRNA expression of *Isg15*, *Ifit2*, and *Ifih1*. Data represent means ± SEM of three independent experiments. and were analyzed by two-tailed, unpaired Student's *t* test; *P < 0.05; **P < 0.01; ***P < 0.001.

Fig. S7.

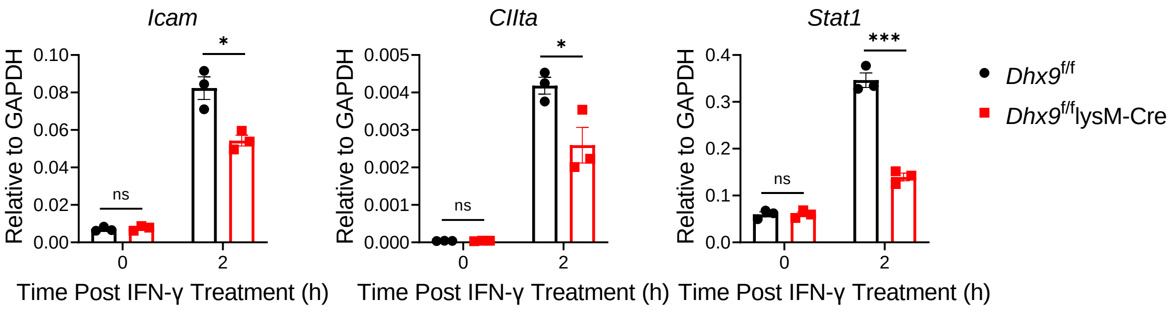

Supplementary Figure 7. The expression levels of IFN-γ inducible ISGs in IFNγ stimulated *Dhx9*$^{f/f}$ and *Dhx9*$^{f/f}$ LysM-Cre BMDMs.

RT-qPCR analysis the expression of *Icam*, *CIIta*, and *Stat1* from *Dhx9*$^{f/f}$ and *Dhx9*$^{f/f}$ LysM-Cre BMDMs stimulated with IFN-γ (100 U/mL) for 2 h. Data represent means ± SEM of three independent experiments, and were analyzed by two-tailed, unpaired Student's t test; $*P < 0.05$; $**P < 0.01$; $***P < 0.001$.

Fig. S8.

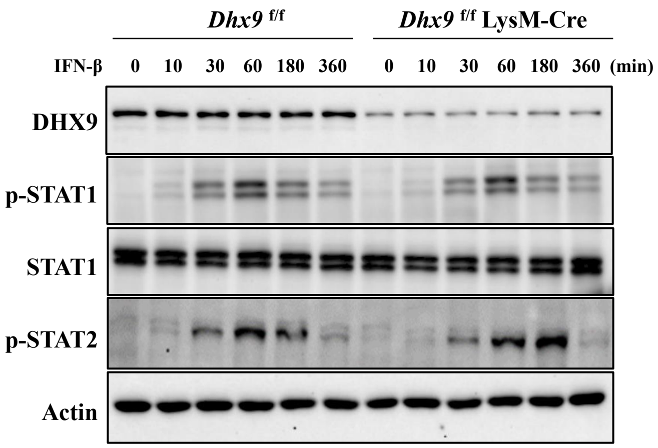

Supplementary Figure 8. The phosphorylation of STAT1 at Tyr701 is not affect in *Dhx9*$^{f/f}$ LysM-Cre BMDMs. Immunoblot analysis of pY-STAT1 and pY-STAT2 in BMDMs from Dhx9$^{f/f}$ and Dhx9$^{f/f}$ LysM-Cre mice stimulated with mouse IFN-β (250 U/mL) for the indicated times. Data are shown as a representative result of three independent experiments

Fig. S9.

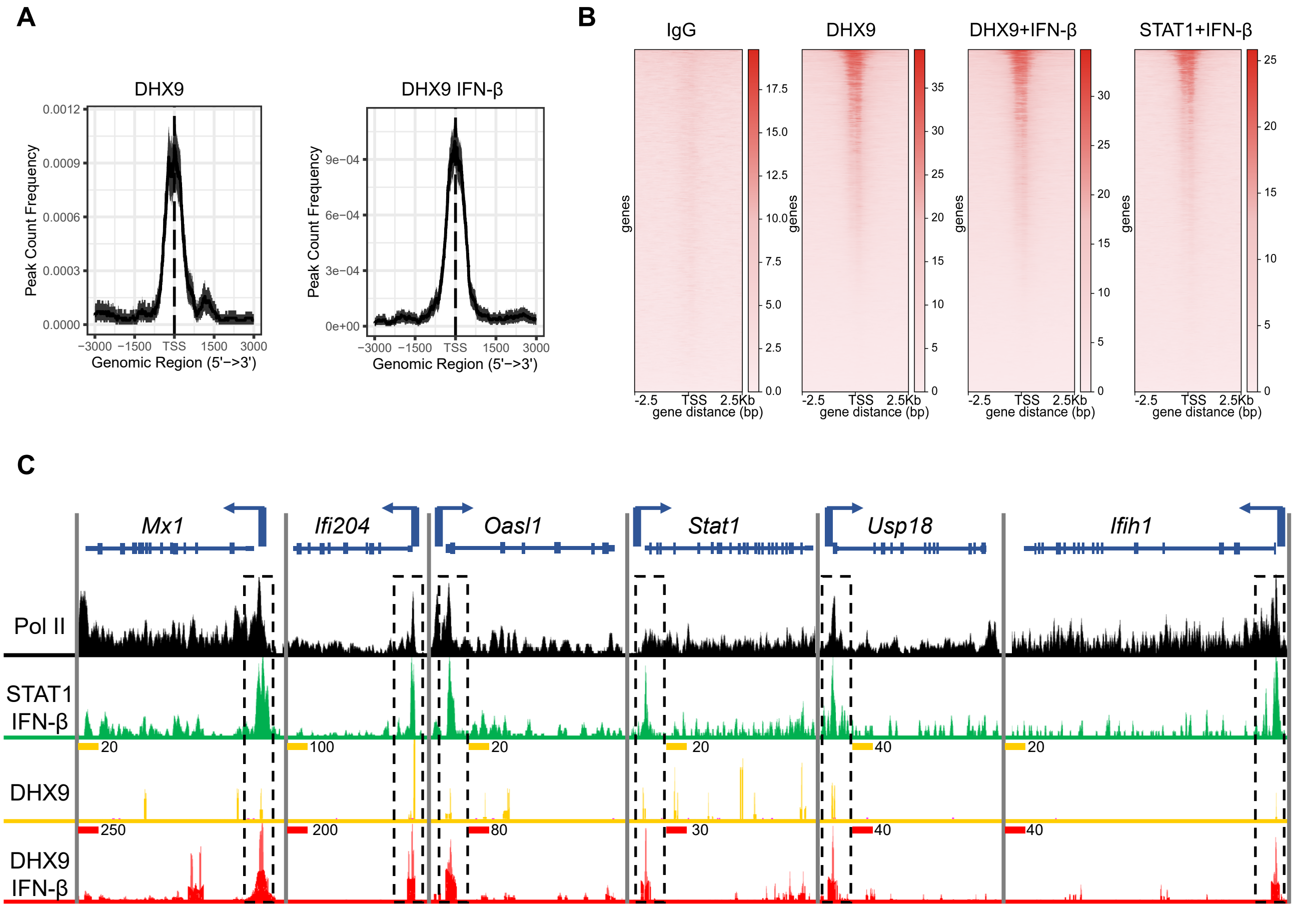

Supplementary Figure 9. IFN-β-induced the recruitment of DHX9 and STAT1 to the promoters of ISGs.

(A) Cut&Tag ChIP analysis of BMDMs stimulated with IFN-β (250 U/mL) or PBS vehicle control for 2 h. The peak count frequency of DHX9 ChIP-seq peaks binding around transcription start site (TSS) regions in 3.0 kb up or downstream of BMDMs.

(B) Heatmaps were used to plot the ChIP-seq signal in 2.5 kb up- or downstream of the TSS of IgG, DHX9, DHX9+IFN-β, STAT1+INFβ in BMDMs.

(C) UCSC genome browser tracks showing ChIP-seq of Pol II, STAT1 (stimulated by INF-β), and DHX9 (with or without INF-β stimulation) bound promoter region of ISGs in BMDMs. The Pol II ChIP-Seq raw data were downloaded from GEO (accession no. GSE106706).

Fig. S10.

A

Rank	Motif	P-value	log P-pvalue	% of Targets	% of Background	STD(Bg STD)	Best Match/Details
1	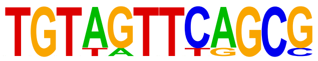	1e-12	-2.936e+01	1.42%	0.00%	51.9bp (0.0bp)	RUNX1(Runt)/Jurkat-RUNX1-ChIP-Seq(GSE29180)/Homer(0.586)
2	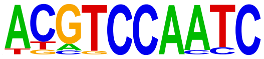	1e-11	-2.686e+01	3.40%	0.17%	57.0bp (70.7bp)	NFYC/MA1644.1/Jaspar(0.756)
3	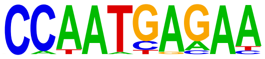	1e-11	-2.586e+01	4.53%	0.43%	56.8bp (64.4bp)	NFYC/MA1644.1/Jaspar(0.806)
**4**	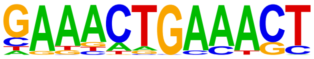	**1e-11**	**-2.564e+01**	**3.68%**	**0.24%**	**46.7bp (55.2bp)**	**ISRE(IRF)/ThioMac-LPS-Expression(GSE23622)/Homer(0.945)**
5	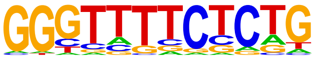	1e-10	-2.492e+01	6.52%	1.09%	50.9bp (61.4bp)	NFkB-p65(RHD)/GM12787-p65-ChIP-Seq(GSE19485)/Homer(0.670)
6	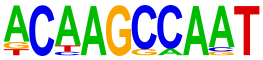	1e-10	-2.480e+01	3.40%	0.20%	61.7bp (63.7bp)	NFY(CCAAT)/Promoter/Homer(0.736)

B

Rank	Motif	P-value	log P-pvalue	% of Targets	% of Background	STD(Bg STD)	Best Match/Details
1	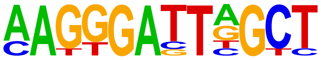	1e-16	-3.731e+01	4.48%	0.05%	41.7bp (69.9bp)	PH0035.1_Gsc/Jaspar(0.724)
2	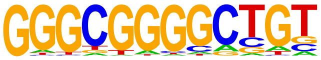	1e-14	-3.419e+01	24.22%	7.13%	55.9bp (62.3bp)	Sp2(Zf)/HEK293-Sp2.eGFP-ChIP-Seq(Encode)/Homer(0.888)
3	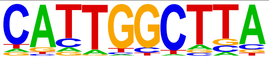	1e-12	-2.898e+01	21.08%	6.30%	53.5bp (62.9bp)	NFYB/MA0502.2/Jaspar(0.772)
4	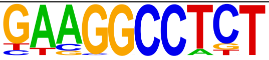	1e-11	-2.568e+01	6.73%	0.58%	49.1bp (64.2bp)	ZFX(Zf)/mES-Zfx-ChIP-Seq(GSE11431)/Homer(0.773)
5	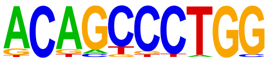	1e-10	-2.520e+01	10.31%	1.72%	56.3bp (57.4bp)	ZFX(Zf)/mES-Zfx-ChIP-Seq(GSE11431)/Homer(0.646)
6	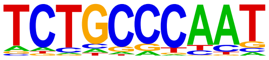	1e-10	-2.420e+01	16.59%	4.71%	54.0bp (60.1bp)	PB0133.1_Hic1_2/Jaspar(0.738)

Supplementary Figure 10. ***De novo*** **motif analysis of DHX9-enriched peaks.**

(A) *De novo* motif analysis of DHX9-enriched peaks (upon IFN-β stimulation) by Homer, showing the top six enriched motifs.

(B) *De novo* motif analysis of DHX9-enriched peaks (under steady state) by Homer, showing the top six enriched motifs.

Table S1. The qPCR primers sequence used in this study

RT-qPCR primers	Sequence (5'-3')
EMCV-Forward	CCTCTTAATTCGACGCTTGAA
EMCV-Reverse	GGCAAGCATAGTGATCGAAG
RHV-1-Forward	GGCTGTGTCATCTGCGAGCA
RHV-1-Reverse	CGACGAAGTCTATATGGTGGGC
MNV-1-Forward	GACCGAGACCACCAAGACTG
MNV-1-Reverse	CACCAAGTCAAGAGAGGCCG
MNV-3-Forward	CAGGAACGCTCAGCAGTCT
MNV-3-Reverse	ATGGGGACGGCCTGTTCAAC
IAV-Forward	GGACTGCAGCGTAGACGCTT
IAV-Reverse	CATCCTGTTGTATATGAGGCCCAT
Isg15-Forward	GGTGTCCGTGACTAACTCCAT
Isg15-Reverse	TGGAAAGGGTAAGACCGTCCT
Oasl2-Forward	TTGTGCGGAGGATCAGGTACT
Oasl2-Reverse	TGATGGTGTCGCAGTCTTTGA
Gbp10-Forward	CTGTGCAGTCTCAAACCAAG
Gbp10-Reverse	CACAAGTCGTTCTTAGG
Ccl5-Forward	GCTGCTTTGCCTACCTCTCC
Ccl5-Reverse	TCGAGTGACAAACACGACTGC
Cxcl10-Forward	GCCGTCATTTTCTGCCTCAT
Cxcl10-Reverse	GCTTCCCTATGGCCCTCATT
Rig-1-Forward	AGCCAAGGATGTCTCCGAGGAA
Rig-1-Reverse	ACACTGAGCACGCTTTGTGGAC
Ifit1-Forward	CAAGGCAGGTTTCTGAGGAG
Ifit1-Reverse	TGAAGCAGATTCTCCATGACC
Mx1-Forward	AACCCTGCTACCTTTCAA
Mx1-Reverse	AAGCATCGTTTTCTCTATTTC
Gapdh-Forward	TGAGGCCGGTGCTGAGTATGTCG
Gapdh-Reverse	CCACAGTCTTCTGGGTGGCAGTG
DHX9-Forward	GCCAATTTCTGGCCAAAGCA
DHX9-Reverse	CGAGGCTCAATGGGGAGTTT
Icam-Forward	TTCACACTGAATGCCAGCTC
Icam-Reverse	CTTCCGTCTGCAGGTCATCT
CIIta-Forward	AGGCCTATGCCAACATTGCG
CIIta-Reverse	CCATAGCATGCTCTTCCGGG
Stat1-Forward	GCTGCCTATGATGTCTCGTTT
Stat1-Reverse	TGCTTTTCCGTATGTTGTGCT
hISG15-Forward	CGCAGATCACCCAGAAGATCG
hISG15-Reverse	TTCGTCGCATTTGTCCACCA
hIFIT2-Forward	AAGCACCTCAAAGGGCAAAAC
hIFIT2-Reverse	TCGGCCCATGTGATAGTAGAC
hIFIH1-Forward	GGCACCATGGGAAGTGATT
hIFIH1-Reverse	ATTTGGTAAGGCCTGAGCTG

Table S2. The ChIP-qPCR primers sequence used in this study

ChiP-qPCR primers	Sequence (5’-3’)
ChiP-*Mx1*-Forward	CACATGCAGTATGGACACCAG
ChiP-*Mx1*-Reverse	GTGTAGCTGGAAGAATCCAGTG
ChiP-*Oasl2*-Forward	GTGCCTCACATCTTTGGGACT
ChiP-*Oasl2*-Reverse	GGCTAGGTTATTCCTGCTTATGC
ChiP-*Ifit3b*-Forward	CTCCAGAGCTAGTCACATGGAC
ChiP-*Ifit3b*-Reverse	CAGTCTCTAGTGCACTCTGCAG
ChiP-*Gbp9*-Forward	CACTGTGGAAAGAGTTTACAT
ChiP-*Gbp9*-Reverse	CTGAGAGAAGCACACGTGCA
